# Harvester perceptions of pesticide impacts on snail collection along Cameroon’s Atlantic coast

**DOI:** 10.1371/journal.pone.0351962

**Published:** 2026-06-18

**Authors:** Yannick Elysée Ewane Epée, Annick Niquaise Enangue Njembele, Christian Bernard Kaldjob Mbeh, Emmanuel Ndinga Go’o, Alexis Hamdja Ngoniri, Kingsley Agbor Etchu

**Affiliations:** 1 Njombe Multipurpose Agricultural Research Station, Institute of Agricultural Research for Development (IRAD), Njombe, Cameroon; 2 Specialized Research Station for Marine Ecosystems, Institute of Agricultural Research for Development (IRAD), Kribi, Cameroon; 3 Mbalmayo Agricultural Research Centre, Institute of Agricultural Research for Development (IRAD), Mbalmayo, Cameroon; 4 National Institute of Cartography (INC), Yaoundé, Cameroon; 5 Nkolbisson Headquarter, Institute of Agricultural Research for Development (IRAD), Nkolbisson, Yaoundé, Cameroon; Hainan University, CHINA

## Abstract

The coastal regions of Cameroon comprising South-West, Littoral, and South are major agricultural hubs and important zones for harvesting African giant land snails (*Achatinidae*). Snail collection provides both a vital protein source for local consumers and a source of income for harvesters. Agricultural intensification has increased pesticide use in these areas, raising concerns about potential impacts on snail populations and human health. This study assessed harvesters’ perceptions of how pesticides influence the quantity and size of snails collected. A total of 211 harvesters from six localities (Kribi, Buea, Limbe, Mbanga, Njombe-Penja, and Dibamba) were surveyed through interviews and participant observations. Descriptive statistics and regression models were applied to explore socio-demographic influences on perceptions. Results showed that while 58% of harvesters believed pesticides reduce snail size, 61% did not perceive an effect on snail quantity. Snail collection was reported to be most successful during nocturnal periods and in October, aligning with ecological seasonality. Regression analysis revealed a weak but statistically significant association between perceptions of pesticide impacts and reported harvesting quantities (*R² = 0.065*), with perceived effects on snail quantity emerging as the only significant predictor. Despite awareness of pesticide risks, 63% of respondents expressed no interest in snail farming, citing preference for wild harvesting. This study highlights local knowledge on pesticide risks but also reveals a gap between awareness and adaptive practices. Findings are framed as perceptions, not direct ecological measurements, and they underline the need for awareness campaigns, sustainable harvesting practices, and supportive policies for alternative snail production systems.

## Introduction

Cameroon’s 402 km Atlantic coastline [1], comprising the Littoral, South-West, and South regions, represents one of the country’s major agricultural production zones. The area hosts large agro-industrial plantations such as the Cameroon Development Corporation (CDC), Plantation du Haut Penja (PHP), and Société Camerounaise de Palmeraies (SOCAPALM), alongside numerous smallholder farms. The humid tropical climate, dense vegetation, and fertile soils of these coastal regions also provide favorable habitats for African giant land snails (Achatinidae) [2,3], which are widely valued as a source of animal protein and income [4–9].

Consumption of snail meat is historically high in the South-West Region of Cameroon, where giant African land snails constitute an important component of local food culture [8,9]. In recent years, however, population displacements and internal migration associated with sociopolitical tensions in some regions of the country have contributed to the spread of snail consumption into major urban centers and communities where these species were not traditionally consumed. This growing demand has increased pressure on existing snail supply systems, which remain heavily dependent on wild harvesting from forests, farms, and peri-urban environments. Snail harvesting therefore serves both subsistence and commercial purposes, providing food for households while also generating income for collectors and traders.

Beyond their nutritional importance, giant African land snails are also associated with medicinal and cultural values in several African communities [10–12]. Despite increasing demand, supply remains largely dependent on wild collection, particularly in agricultural landscapes where snails are abundant.

At the same time, agricultural intensification in coastal Cameroon has been accompanied by increasing pesticide use in both industrial plantations and smallholder farming systems [13–19]. This situation raises concerns regarding potential impacts on terrestrial snail populations, which frequently feed on vegetation and organic matter exposed to agricultural chemicals [20–23]. In addition, pesticide contamination may potentially represent a public health concern for consumers through the ingestion of contaminated snail meat [19–28].

Previous studies have demonstrated that pesticide residues can accumulate in snail tissues and negatively affect growth, reproduction, survival, and physiological health [22,29–31]. However, the ecological effects of pesticides are not always directly observable under field conditions, and local perceptions of environmental risks may vary according to harvesters’ experiences, environmental knowledge, and socio-economic conditions.

In the present study, the term perception refers to harvesters’ observations and interpretations regarding possible changes in the quantity (abundance) and size characteristics of snails collected from environments potentially exposed to pesticides. These perceptions include observations of reduced snail abundance, smaller snail size, or concerns regarding environmental contamination. In rural areas where ecological monitoring systems remain limited, harvesters’ observations may provide important preliminary indications of environmental change and resource decline.

Recent experimental studies conducted in Cameroon showed that some pesticides commonly used in agriculture, including glyphosate, cypermethrin, and metalaxyl, can negatively affect the growth and health of giant African land snails under controlled conditions [22,29]. Nevertheless, no previous perception-based study has investigated whether snail harvesters along Cameroon’s Atlantic coast associate pesticide use with changes in snail availability or quality under field conditions.

Therefore, the present study aimed to assess whether the impacts previously identified experimentally are also perceived in the field by snail harvesters. Specifically, the study explored perceptions among both subsistence and commercial collectors regarding the effects of pesticide use on snail quantity and size, while also documenting harvesting practices and attitudes toward snail farming as a potential alternative to wild collection. The findings contribute to understanding the socio-economic dimensions of pesticide risk perception and may support future initiatives related to sustainable harvesting, environmental awareness, public health, and alternative snail production systems in Cameroon.

## Materials and methods

### Description of study area

**Fig 1:**
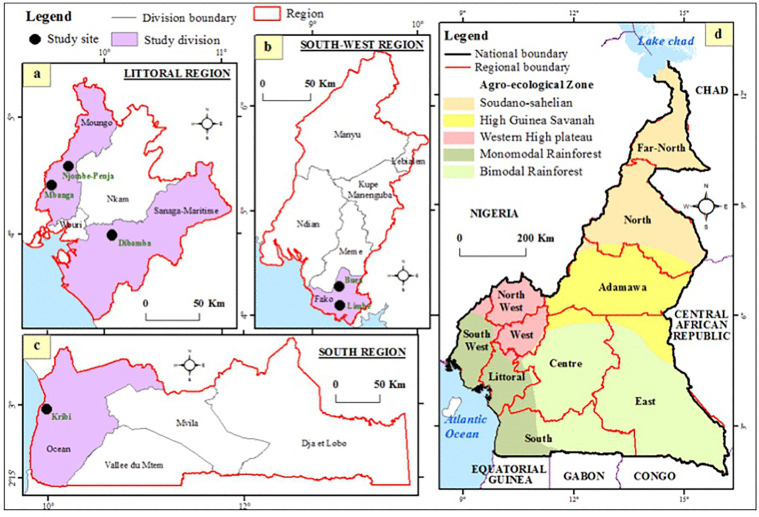
Map of the study area. Study sites were located in three regions along Cameroon’s Atlantic coast Kribi (South Region), Buea and Limbe (South-West Region), and Mbanga, Njombe-Penja, and Dibamba (Littoral Region). The map was produced in ArcGIS from GPS coordinates collected in the field and OpenStreetMap geographic data ( ; OpenStreetMap contributors, ODbL licence).

The study was conducted in three coastal regions of Cameroon (Littoral, South, and South-West) at six sites: Kribi (South), Buea and Limbe (South-West), and Mbanga, Njombe-Penja, and Dibamba (Littoral) (Fig 1). Sites were purposively selected because they combine intensive agricultural activities with high snail harvesting, making them representative of pesticide-exposed environments. All fall within the humid forest agroecological zone, characterized by a monomodal rainfall pattern with one long rainy season and one dry season. The volcanic soils of Mount Cameroon (Fako) and Mount Manengouba (Moungo) contribute to high agricultural productivity, which in turn supports diverse snail populations. The major agro-industrial companies present in the area are CDC (Buea, Limbe), SOCAPALM (Kribi), and PHP (Njombe-Penja). The sites also represent a range of urban and peri-urban contexts, with populations from around 5,000 in Dibamba to over 130,000 in Buea. This diversity enabled the study to capture variation in harvesting practices and socio-demographic characteristics among respondents.

### Data collection and sampling

A total of 211 snail harvesters participated in the study (Table 1). Study sites were purposively selected because they combine intensive agricultural activities with important snail harvesting practices, making them representative of environments potentially exposed to pesticides. Participants were recruited using two complementary approaches. In Njombe-Penja and Mbanga, recruitment was conducted directly in local markets where snails are commonly sold. In other localities, participants were identified through community nomination and referrals from local residents familiar with harvesting activities. This combined strategy facilitated access to experienced harvesters actively involved in snail collection. Data collection combined semi-structured interviews and participant observations. Interviews documented socio-demographic characteristics (age, gender, marital status, education level, household size, and occupation), harvesting practices (collection sites, periods, seasonality, and harvesting experience), and perceptions regarding possible pesticide effects on snail quantity and size. Additional questions explored awareness of pesticide use and willingness to engage in snail farming as an alternative source of production. Participant observations complemented interviews by documenting harvesting activities and environmental conditions during collection practices. Because purposive and referral-based sampling approaches were used, the findings may not fully represent all snail harvesters across Cameroon’s coastal regions. However, the approach was considered appropriate for accessing knowledgeable participants involved in harvesting activities.

**Table 1:** Distribution of snail collectors across study localities.

Localities	Number of snail collectors	Percentage
Limbe	50	23.7
Buea	30	14.2
Dibamba	30	14.2
Njombe-Penja	35	16.6
Mbanga	30	14.2
Kribi	36	17.1
**Total**	**211**	**100**

Number and proportion of respondents surveyed in each study site along Cameroon’s Atlantic coastal regions (South, South-West, and Littoral).

The Linear Regression Model formula used for this study is: Coll = Y (S1, S2, S3, S4, S5, S6, S7, S8, S9, S10, S11, S12, S13, S14, S15, S16, S17, S18, ε)

See Table 2 for the description of codes used.
Coll = Collection
S1 = Sex (Gender of respondent).
S2 = Age (Age in year).
S3 = Marstat (Marital status).
S4 = Householdsize (Size of the household).
S5 = Edulevel (Level of education).
S6 = Mainact (Main activity).
S7 = Region (Region of origin).
S8 = Ethnic (Ethnic group).
S9 = collExp (Experience of collection)
S10 = Collectzone (Zone of collection).
S11 = Bestzone (Best Place of snails’ collection).
S12 = Quantity (Quantity of snail collected).
S13 = Month (Month of collection).
S14 = Season (Season of collection).
S15 = Time (Time of day when harvesting occurs).
S16 = PestiQty (Perception on pesticide use on snail Quantity).
S17 = PestiSnailSize (perception on pesticide use on snail size).
S18 = Snailbreeder (Consideration of snail breeding).
ε = Disturbance

**Table 2:** Description of main socio-economic variables.

Parameter	Description	code
Sex	Gender of respondent	1-Male; 2-Female
Age	Age in year	1- < 30; 2-30-40; 3- 40-50; 4-50-60; 5-> 60
Marstat	Marital status	1-Single; 2-Married; 3-Separated; 4- Divorced; 5-Widow
Household size	Size of the household	Number of people in the house
Edulevel	Level of education	1-Primary; 2-Secondary; 3-Tertiary
Mainact	Main activity	1-Farming; 2-Trading; 3-Civil servant; 4-Artisan; 5-Other
Region	Region of origin	Name of the region
Ethnic	Ethnic group	Name of the tribe
collExp	Experience of collection	Years of harvesting experience
Collectzone	Harvesting location	1-Within the village; 2-Out of the village
Bestzone	Preferred harvesting location	1-Around houses; 2-Around garbage; 3-Around toilet; 4-Around farms; 5-In the forest; 6-others
Quantity	Quantity of snail collected	Number of 10L buckets collected
Month	Month of collection	Name of the month
Season	Season of collection	Name of the season
Time	Time of day when harvesting occurs	1-Morning; 2-Afternoon; 3-Night
PestiQty	Perception on pesticide use on snail quantity	1-Yes; 2-No
PestiSnailSize	Perception on pesticide use on snail size	1-Yes; 2-No
Snailbreeder	Consideration of snail breeding	1-Yes; 2-No

This table presents the socio-economic parameters assessed in the study, along with their definitions and the numerical codes assigned for statistical analysis.

### Statistical analysis

Data were analyzed using descriptive and inferential statistical methods. Descriptive statistics summarized respondents’ socio-demographic characteristics, harvesting practices, and perceptions of pesticide impacts. Chi-square tests were used to evaluate associations between categorical variables, including perceptions of pesticide impacts and socio-demographic characteristics. Welch’s t-test compared mean harvesting quantities between male and female respondents. Harvesting quantities were estimated using the standard 10L bucket commonly used by local collectors. ANOVA and Kruskal–Wallis tests were applied to assess differences in harvesting quantities across demographic and geographic groups. Correlation and linear regression analyses were conducted to explore the relationship between harvesters’ perceptions of pesticide impacts and reported snail collection quantities. Logistic regression was used to assess factors associated with willingness to consider snail farming as an alternative livelihood activity. All analyses were conducted using Stata 12.0, SPSS version 20, and Microsoft Excel 2021. Statistical significance was considered at *p < 0.05*. All statistical outputs were cross-checked using Julius AI (Caesar Labs Inc., February 26 version, 2024) implementing R statistical packages.

### Ethics statement

This study complied with applicable national research ethics guidelines in Cameroon. The research consisted exclusively of voluntary interviews with adult participants and did not involve biological sampling, medical interventions, or collection of identifiable personal information. Participants were informed about the objectives of the study prior to participation, and verbal informed consent was obtained orally before each interview. Consent was documented through the participant’s voluntary agreement to proceed with the questionnaire. This procedure was considered appropriate for this minimal-risk, interview-based study conducted in community settings. According to national regulations governing non-interventional socio-economic surveys involving consenting adult participants, formal institutional ethics approval was not required for this study. Only adults aged 21 years and above participated in the study. No endangered or protected animal species were involved, and the snails discussed (*Achatinidae*) are common non-threatened species.

## Results

### Socio-economic characteristics of respondents

**Fig 2:**
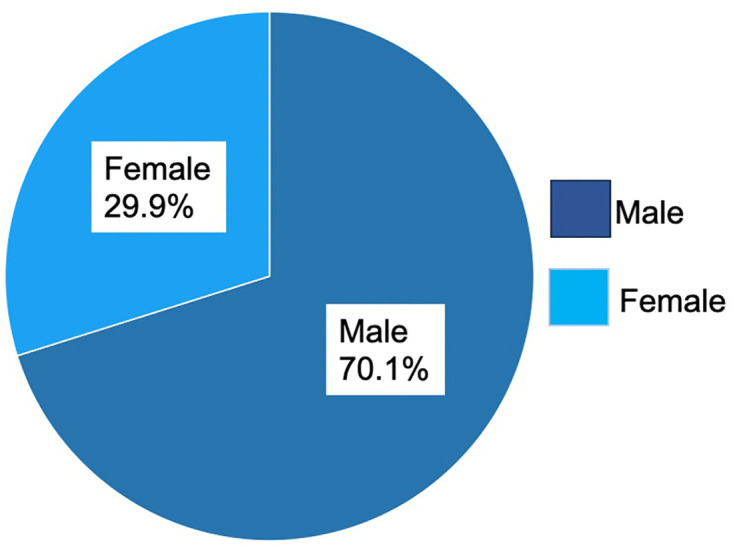
Gender distribution of snail collectors. The majority of respondents were male (70%), while females represented 30% of the total sample.

**Fig 3:**
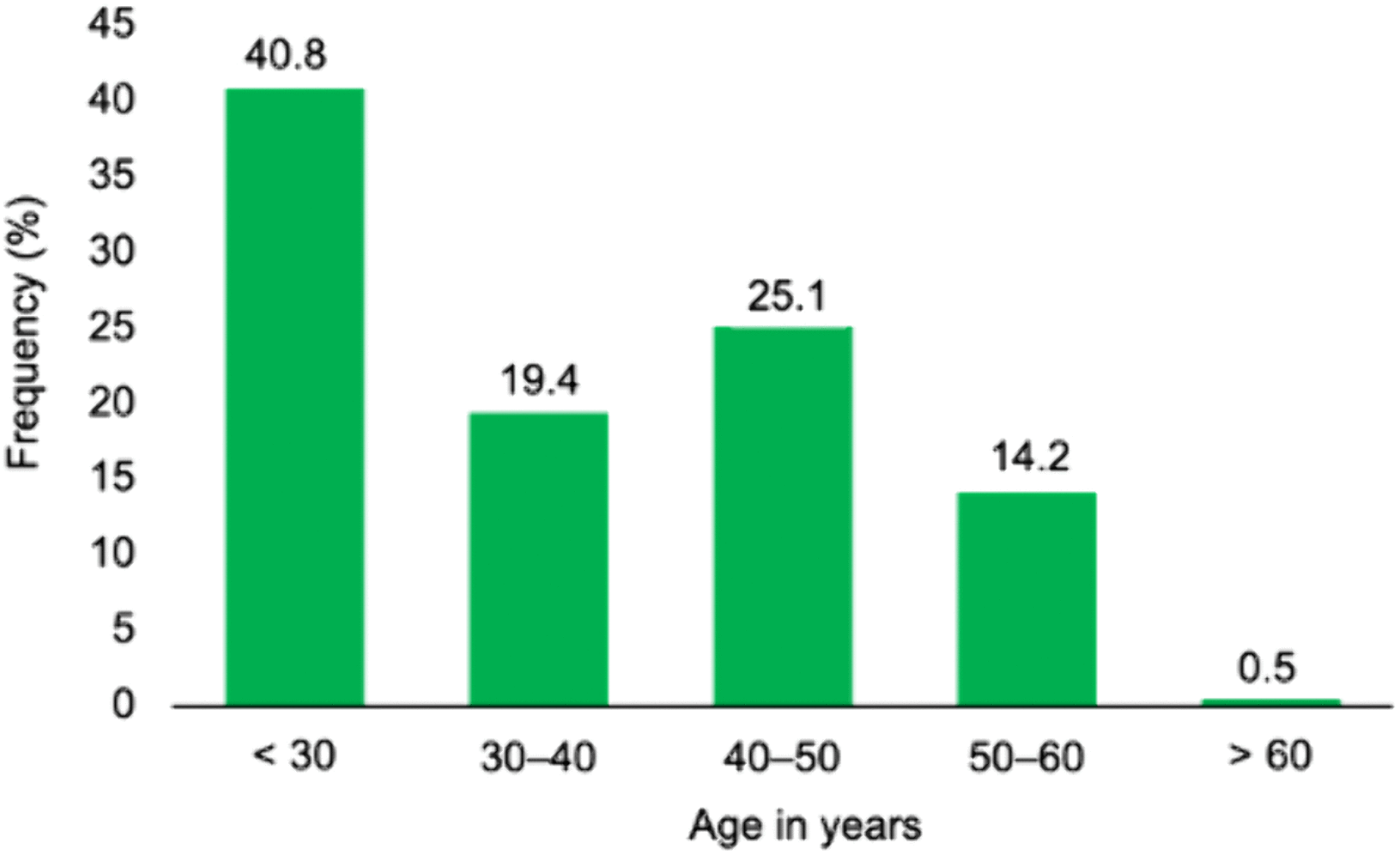
Age distribution of respondents. Most collectors were under 30 years of age, with fewer participants above 50 years.

**Fig 4:**
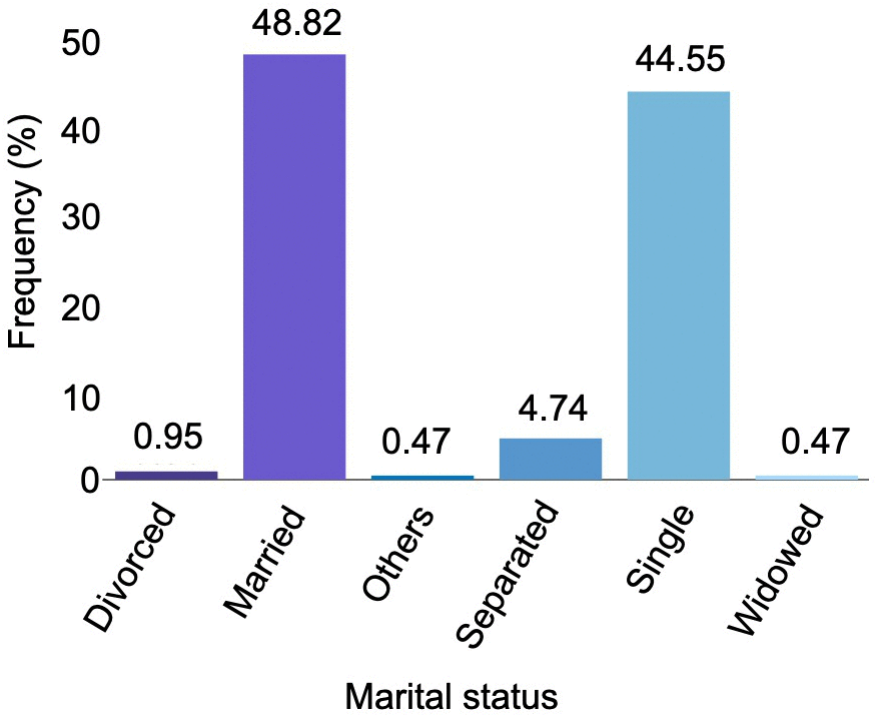
Marital status of respondents. Nearly half of respondents were married (49%), while 45% were single.

**Fig 5:**
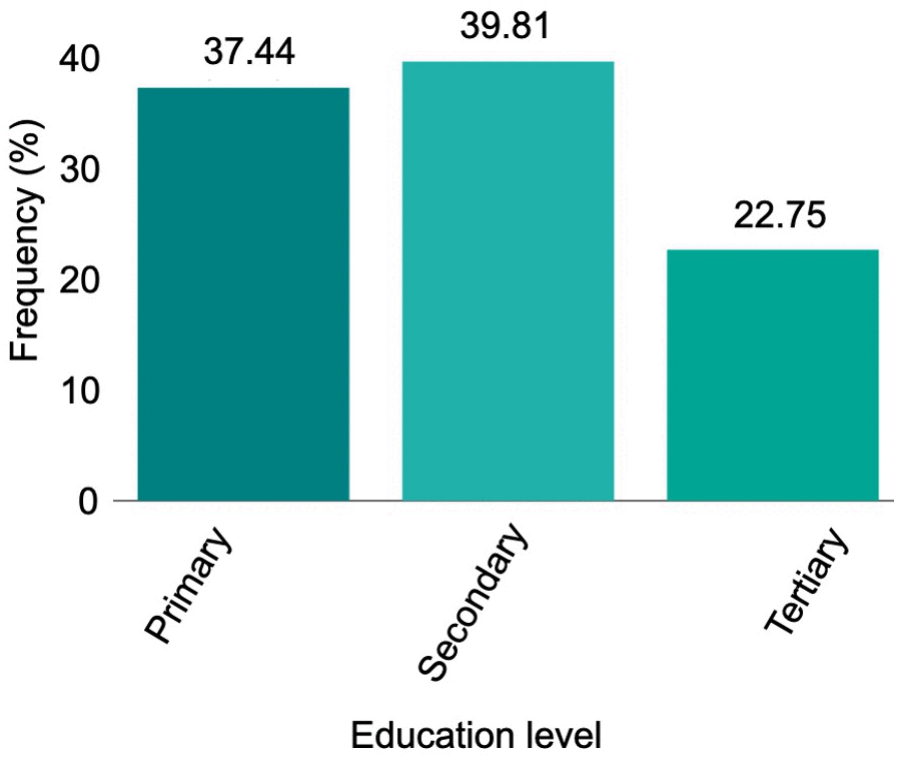
Educational level of respondents. Most collectors had completed secondary education (approximately 40%), and 23% had tertiary education.

**Fig 6:**
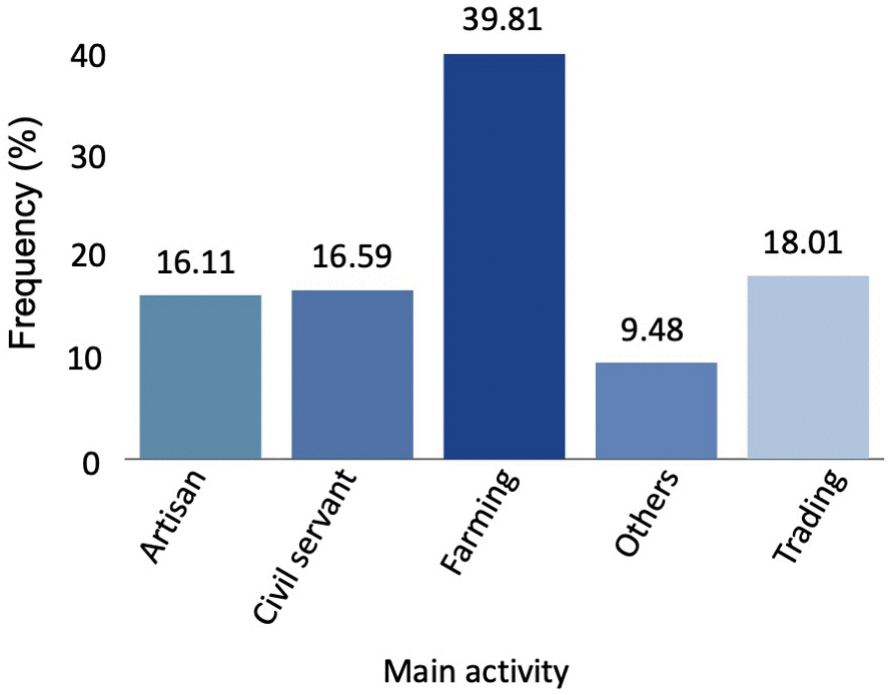
Primary occupation of respondents. Farming was the predominant occupation, representing 40% of the sample population.

**Fig 7:**
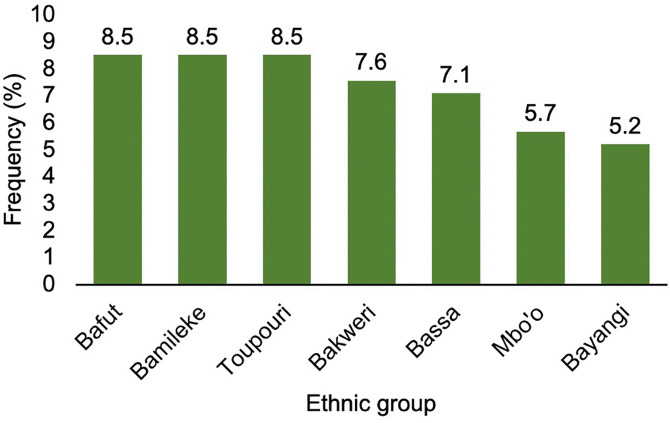
Representation of the top seven ethnic groups among respondents. A total of 47 ethnic groups were identified among respondents. The histogram shows the percentage representation of the seven most frequent groups, with Bafut, Bamileke, and Toupouri being the most represented.

Among the 211 respondents, 70% were male and 30% female (Fig 2). Most respondents were under 30 years old (41%), while only 0.5% were over 60 years old (Fig 3). Nearly half were married (49%), whereas 45% were single (Fig 4). Education levels were relatively high, with 40% having completed secondary school and 23% tertiary education (Fig 5). Farming was the most common primary occupation (40%) (Fig 6). The sample was ethnically diverse, comprising 47 ethnic groups. Bafut, Bamileke, and Toupouri each represented approximately 8–9% of respondents, followed by Bakweri (8%) (Fig 7). On average, harvesters had seven years of harvesting experience.

### Harvesting practices and collection perceptions

**Fig 8:**
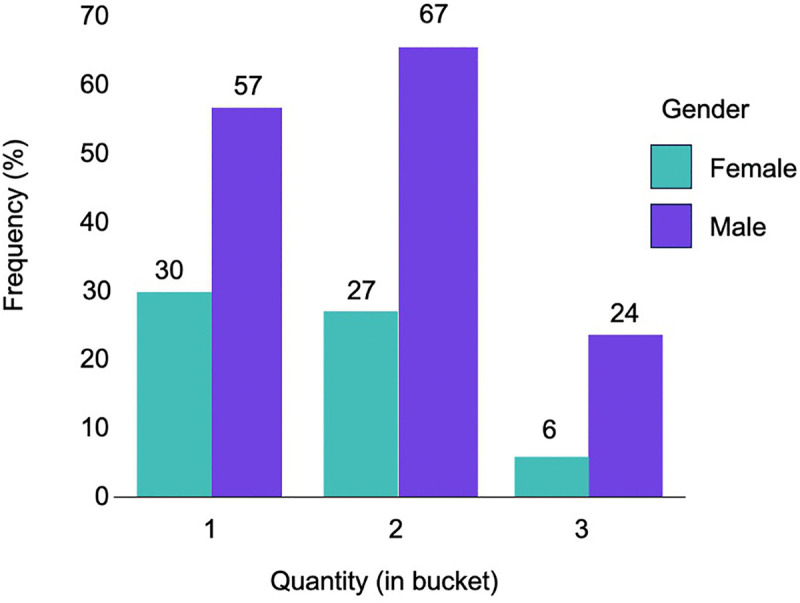
Average quantity of snails collected according to gender. Male respondents collected slightly higher quantities of snails than female respondents (1.78 ± 0.71 *vs.* 1.61 ± 0.66).

**Fig 9:**
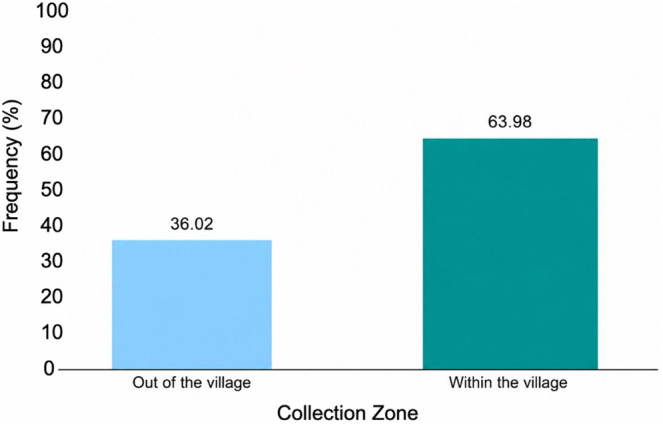
Distribution of snail collection sites within and outside villages. Most snail collections were recorded within villages (64%), whereas harvesting outside villages represented 36% of the reported collection sites.

**Fig 10:**
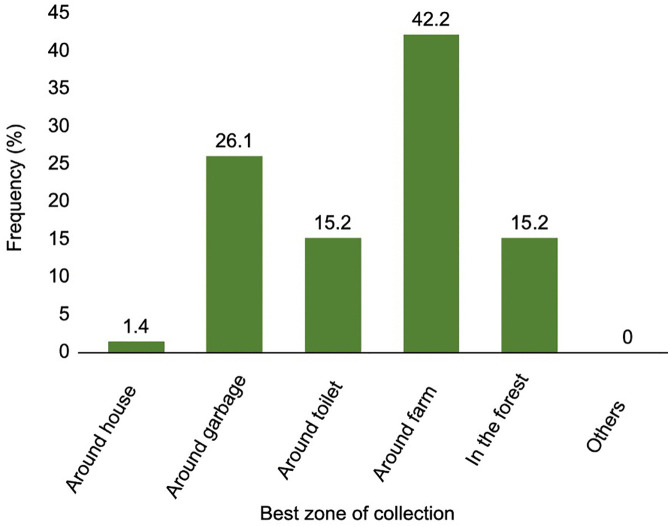
Frequency of snail harvesting according to collection zones. Farms were the principal harvesting zones (42%), followed by waste disposal areas (26%), while collection near houses remained minimal (1%).

**Fig 11:**
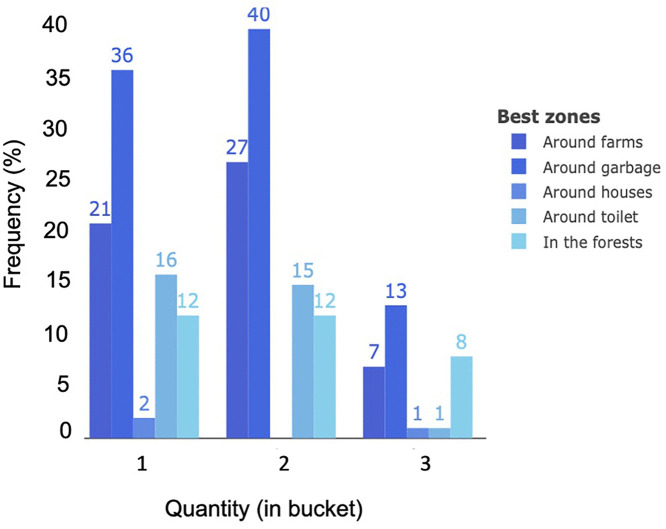
Distribution of harvesting quantities across collection zones. Across the main harvesting zones, most collectors reported gathering one to two 10L buckets of snails per session, while three-bucket collections were less frequent.

**Fig 12:**
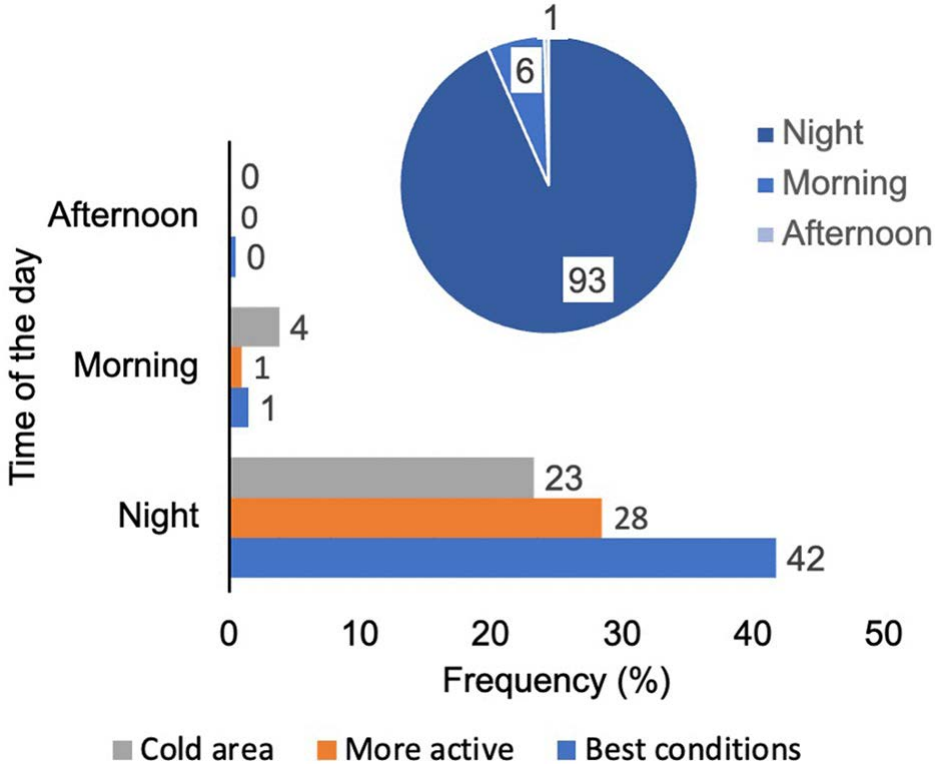
Preferred harvesting periods and reasons for night harvesting. Harvesting occurred mainly at night (93%), consistent with the nocturnal activity of giant African land snails. Increased snail activity and favorable humidity were cited by 42% of respondents as the main reasons for night-time collection.

**Fig 13:**
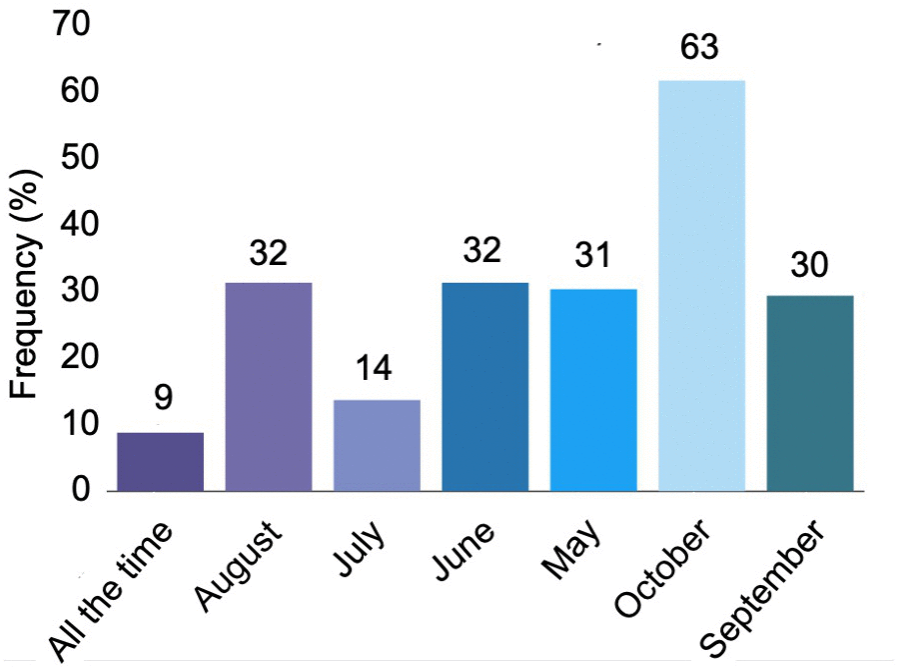
Monthly variation in snail harvesting success. Harvesting activity peaked in October (63%) and was lowest in July (14%), reflecting seasonal changes in snail activity and environmental conditions.

**Fig 14:**
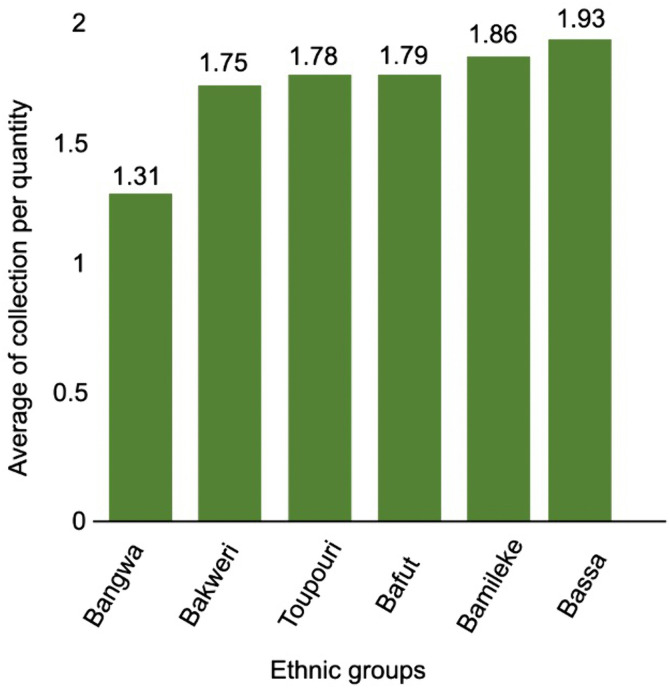
Average harvesting quantity according to ethnic group. Among respondents, the Bassa (1.96 buckets), Bamileke (1.86 buckets), and Bafut (1.73 buckets) ethnic groups reported the highest average harvesting quantities.

Most respondents collected one to two 10L buckets of snails per harvesting session, although some reported harvesting up to three buckets. Two-bucket collections were the most common harvesting category among both men and women. Male collectors harvested slightly more buckets on average than female collectors (1.78 ± 0.71 *vs.* 1.61 ± 0.66) (Fig 8). Most snail collections were recorded within the village (64%), whereas collections outside the village represented 36% of the total sampling sites (Fig 9). Village farms were the principal harvesting sites (42%), followed by waste disposal areas (26%), whereas collection near houses remained minimal (1%) (Figs 10 and 11). Harvesting was predominantly nocturnal (93%), with collectors being over 15 times more likely to harvest snails at night than during the morning (Fig 12). Night-time harvesting was preferred because of higher snail activity and favorable humidity conditions (42%) (Fig 12). Seasonal variation strongly influenced harvesting success: October recorded the highest yields (63%), whereas July showed the lowest (14%) (Fig 13). Ethnic differences in harvesting quantities were modest, with Bassa and Bamileke respondents reporting slightly higher averages (1.9 buckets) compared with other groups (Fig 14).

### Perceptions of pesticide impacts and attitudes toward snail farming

**Fig 15:**
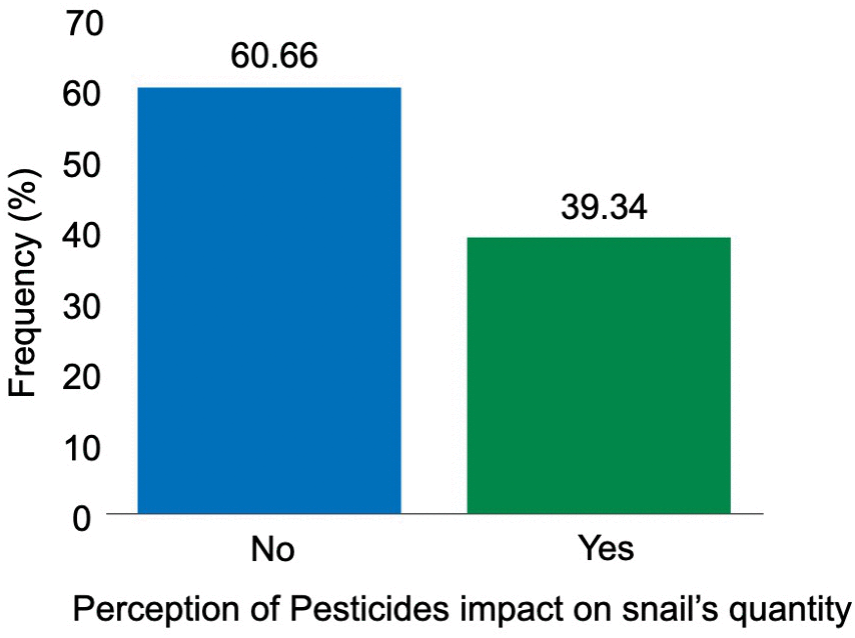
Perceptions of pesticide effects on snail quantity. Approximately 61% of respondents believed that pesticide use did not affect the number of snails collected.

**Fig 16:**
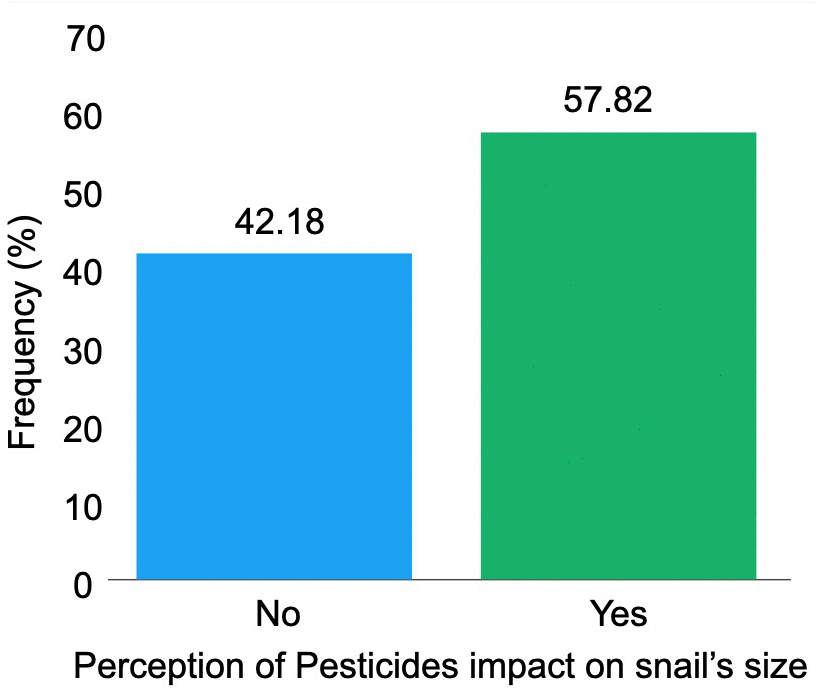
Perceptions of pesticide effects on snail size. Nearly 58% of respondents reported that pesticide use reduced the size of collected snails.

**Fig 17:**
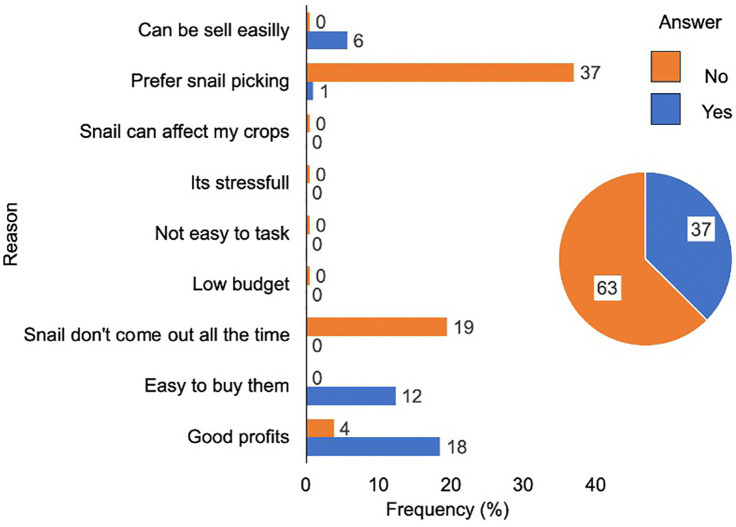
Willingness to engage in snail farming. Approximately 63% of respondents expressed unwillingness to adopt snail farming, mainly because wild harvesting was considered easier and less demanding.

Respondents reported mixed views regarding pesticide impacts. Approximately 58% believed that pesticide use reduced snail size, whereas 61% considered that pesticide use did not affect the quantity of snails collected (Figs 15 and 16). Despite awareness of potential pesticide risks, 63% of respondents expressed no interest in snail farming, citing a preference for wild harvesting. Among respondents interested in snail farming (18%), profitability was identified as the principal motivation (Fig 17).

### Statistical analysis

Across all harvesting categories, the proportion of male collectors (70%) was significantly higher than that of female collectors (30%) (*χ²* test, *p < 0.001*). No significant differences in harvesting rates were observed across localities (*p = 0.39–0.78*), education levels (*p = 0.08*), or ethnic groups (*p > 0.40*). Although male collectors harvested slightly more buckets on average than female collectors (1.78 ± 0.71 vs. 1.61 ± 0.66), this difference was not statistically significant (*p = 0.111*). Perceptions of pesticide impacts showed no significant difference between beliefs regarding effects on snail size and quantity (*p = 0.77*). A weak positive correlation was observed between perceptions of pesticide impacts and harvesting quantity (*r = 0.21*). Although the linear regression model was statistically significant (*p = 0.033*), it explained only a limited proportion of the observed variation (*R² = 0.065*). The only significant predictor was the perception that pesticides reduce snail quantity (*p = 0.009*). Logistic regression analyses identified no significant socio-demographic predictors of willingness to engage in snail farming, although weak non-significant trends suggested slightly lower interest among middle-aged respondents and marginally higher interest among respondents with secondary education.

## Discussion

This study explored snail harvesters’ perceptions of pesticide impacts on snail size and quantity in Cameroon’s coastal agricultural zones, highlighting both socio-economic characteristics and local awareness of environmental risks.

Most respondents were men, who tended to collect more snails, reflecting Cameroon’s gendered division of labor, where men often engage in night-time and physically demanding activities. However, women remain active participants in snail harvesting and trade, as noted in previous studies [32,33]. The predominance of young collectors under 40 mirrors Cameroon’s demographic structure [34]. Farming was the main occupation, likely giving collectors easier access to snails in agricultural areas.

Most participants had at least secondary education, which may have facilitated respondents’ understanding of the survey questions. Ethnic diversity among respondents was high, with 47 groups represented consistent with Cameroon’s cultural heterogeneity [34]. Although Bafut, Bamileke, and Toupouri respondents were more frequent, ethnicity did not significantly influence harvest rates. This is notable since snail consumption is typically associated with South-West communities such as the Banyang, Mbo, Balung, and Bakweri [8,9]. The participation of groups like the Bamileke and Bafut may reflect economic motivations, as these communities are known for trade and entrepreneurship [35,36]. The notable participation of the Toupouri is surprising, as they originate from northern Cameroon, where snail consumption is generally limited by cultural norms. Their involvement may reflect adaptation or economic motivation, given that earlier studies report their traditional consumption of another local species, *Archachatina degneri* [8].

Harvesting occurred mainly at night, consistent with the nocturnal behavior of *Achatinidae* snails [37–39] and cultural norms that favor men’s involvement in night-time activities. Farms and waste disposal areas were the main collection sites, likely due to food availability, while collection near houses was minimal. No significant differences were found in collection rates across locations. Harvesting peaked between May and October, aligning with the rainy season when *Achatinidae* snails are most active and abundant [22,37–39].

Perceptions of pesticide impacts varied among respondents. Most harvesters did not associate pesticide use with a reduction in the quantity of snails collected, although many reported observing smaller snail sizes in areas exposed to agricultural chemicals. Because these findings are based on harvesters’ perceptions and self-reported experiences rather than direct ecological measurements or pesticide residue analyses, they should be interpreted cautiously. Statistical analyses revealed only weak associations between perceived pesticide impacts and reported harvesting quantities, suggesting that the observed trends may reflect subjective experiences influenced by local environmental knowledge and livelihood practices. In addition, the low explanatory power of the regression model indicates that other ecological, environmental, and socio-economic factors not assessed in the present study may also contribute to harvesting outcomes. Nevertheless, experimental studies conducted under controlled conditions have shown that several pesticides can negatively affect the growth, reproduction, and physiological health of giant African snails [22,29–31]. Long-term ecological monitoring and residue analyses would therefore be necessary to determine whether comparable effects occur in wild snail populations under field conditions. Although demand for snail meat is increasing, most respondents were reluctant to engage in snail farming, citing the ease of collecting wild snails and the challenges of rearing them. Logistic regression confirmed no significant socio-demographic predictors of willingness to farm snails, though individuals with secondary education showed slightly higher interest. Barriers such as pest attacks, predation, lack of technical knowledge, and limited financial resources likely discourage engagement in snail farming [32,33].

Overall, snail harvesting in coastal Cameroon remains a culturally rooted and economically significant activity. The findings suggest that perceptions of pesticide risk are shaped more by livelihood experience than by measured ecological change, underscoring the importance of integrating local knowledge into sustainable management and public health initiatives.

## Conclusion

This study examined how snail harvesters along Cameroon’s Atlantic coast perceive the effects of pesticide use on snail populations and harvesting practices. Most respondents did not associate pesticides with reduced snail abundance, although many reported observing smaller snails in areas exposed to agricultural chemicals. These findings are based on harvesters’ perceptions and self-reported experiences rather than direct ecological measurements of pesticide contamination or snail population decline. Harvesting practices appeared to be influenced mainly by environmental conditions, livelihood activities, and cultural habits, while socio-demographic factors showed little effect. The study also highlights the importance of local ecological knowledge in areas where environmental monitoring remains limited. Therefore, the findings should be interpreted as perception-based observations rather than direct evidence of pesticide-induced declines in snail populations. Strengthening environmental awareness, encouraging sustainable harvesting practices, and supporting small-scale snail farming through technical and financial assistance could help reduce pressure on wild snail populations while improving rural livelihoods. Future studies combining harvesters’ observations with ecological monitoring and pesticide residue analyses would provide a clearer understanding of the long-term effects of agricultural intensification on terrestrial snail populations in Cameroon.

## Supporting information

S1 File Survey administered to snail harvesters. This file contains the full questionnaire used to collect socio-demographic information, harvesting practices, and perceptions of pesticide impacts among snail harvesters in the study regions.
(DOCX)

S2 File Minimal data set used for statistical analyses. This file contains the anonymized minimal data set used to perform all statistical analyses reported in the manuscript, including variables related to socio-demographic characteristics, harvesting practices, and perceptions of pesticide impacts. The dataset is provided to enable full replication of the study’s findings.
(XLSX)

S3 File Sample of a completed questionnaire. This file provides an anonymized example of a completed questionnaire, illustrating how responses were recorded during field interviews. The verbal consent procedure was conducted orally by the interviewer before administering the questionnaire.
(PDF)
