# Supplementary material for: Harvester perceptions of pesticide impacts on snail collection along Cameroon’s Atlantic coast
Source: PLoS One. 2026 Jun 18;21(6):e0351962. doi: 10.1371/journal.pone.0351962 (PMC13278449; doi:10.1371/journal.pone.0351962)
Supplement: S1 File — This file contains the full questionnaire used to collect socio-demographic information, harvesting practices, and perceptions of pesticide impacts among snail harvesters in the study regions. (DOCX) [file pone.0351962.s001.docx]

**SURVEY ON THE PERCEPTION OF PESTICIDES IMPACT ON AFRICAN GIANTS’ SNAILS’ HARVESTING WITHIN SOME AGRICULTURAL SITES OF CAMEROON COASTAL REGIONS**

**IDENTIFICATION OF SNAIL COLLECTORS**

| Date: /_/_/_/_/ 202 | N^o^ sheet: /_/_/_/  investigator Code: /_/_/_/ | Locality: …………………………  Region: ………………………  country: ……………Cameroon…… |
| --- | --- | --- |
| Coordinator: ………….D**r** ENANGUE N. Annick Niquaise…………………………….  Supervisor: …KALDJOB M. Christian Bernard ………………………………….  Investigator: ………………............................................................................................ | | |

**PART I - CHARACTERIZATION OF THE COLLECTOR**

**P1.01**- **gender**  1- Male ; 2 - Female

**P1.02**- **Age** 1- < 30   ; 2- 30-40  ; 3- 40-50  ;4- 50-60  ; 5- ≥ 70

**P1.03**- **marital status** 1- Single  ; 2- Married  ;3- Separated  ; 4- Divorced ; 5- Widow ; 6- Other …………………

**P1.04**-What is the size of your household?…………………………….

**P1.06- What is your level of education?** 1-Primary  ; 2-Secondary  ; 3- Tertiary

**P1.07- What is your main activity ?**1- Farming ; 2-Trading ; 3- snail collector  ; 4- snail seller ; 5-Others……………..

**P1.9-** **What is your region of origin?** ………………………………….

**P1. 10-** **From** **which ethnic group are you?**…………………………………………….

**P1.11-** **How long have you been collecting snails? …**1- several weeks ; 2- several months. ; 3- several years

**PART II: SNAILS COLLECTION**

**P2.01- Where do you collect snails?** 1- Within the village ; 2- out of the village

**P2.02- If out of the village, please specify the location……………………………………..**

**P2.03- How much snails do you collect (in bucket)?**

………………………………………………………………………………………

**P2.04- At what month do you collect snails the most?**

……………………………………………………………………………………………

**P2.05- Please explain …………………………………………………………………………**

**P2.06- At what season do you collect snails the most?………………………………………**

**P2.07- Please explain …………………………………………………………………………**

**- At what time of the day do you collect snails?**

1. Morning ; 2- Afternoon ; 3- Night

**P2.08- Please explain**……………………………………………………………………

……………………………………………………………………………………………

**P2.09- Where do you collect the most?** 1-Around houses ; 2- Around garbage ; 3- Around toilet ; 4- Around farms ; 5- In the forest ; 6- others

**P2.10- If in the farm, which crops attract them the most?** 1- Tomato ; 2-Maize ; 3- African vegetables ; 4-Others…………………………………………..

**P2.11- If in the plantation, which crops attract them the most?** 1- Banana ; 2- Cocoa ; 3- Palms ; 4- Rubber ; 5- Others

**P2.12- Do you think that the use of agricultural pesticides (fungicides, herbicides, insecticides) affects the population (quantity) of snails present in farms?**

**1 –** Yes ; 2- No

**P2. 13- -If yes explain …………………………………………………….. ………………………………………………………………………………..**

**P2.14- Do you think that the use of agricultural pesticides (fungicides, herbicides, insecticides affect the size of snails in the farm?**

**1 –** Yes ; 2-No

**P2. 15- If yes explain? …………………………………………………….. ……………………………………………………………………………….**

**P2.16- Do you consider yourself breeding snails?** 1 – Yes ; 2-No

**P2.17- If yes explain ..................................................................................................................**

**…………………………………………………………………………………………………**

**P2.18- If no explain ..................................................................................................................**

**…………………………………………………………………………………………………**

**…………………………………………………………………………………………………**
