# Supplementary material for: Harvester perceptions of pesticide impacts on snail collection along Cameroon’s Atlantic coast
Source: PLoS One. 2026 Jun 18;21(6):e0351962. doi: 10.1371/journal.pone.0351962 (PMC13278449; doi:10.1371/journal.pone.0351962)
Supplement: S3 File — This file provides an anonymized example of a completed questionnaire, illustrating how responses were recorded during field interviews. The verbal consent procedure was conducted orally by the interviewer before administering the questionnaire. (PDF) [file pone.0351962.s003.pdf]

MINISTERE DE LA RECHERCHE SCIENTIFIQUE ET DE L'INNOVATION (MINRESI)
MINISTRY OF SCIENTIFIC RESEARCH AND INNOVATION

INSTITUT DE RECHERCHE AGRICOLE POUR LE DEVELOPPEMENT (IRAD)
INSTITUTE OF AGRICULTURAL RESEARCH FOR DEVELOPMENT

QUESTIONNAIRE SUR L'ANALYSE DU RAMASSAGED'ESCARGOTS ACHATINA DANS LA ZONE AGROÉCOLOGIQUE VI CAMEROUN

1) Nom de l'enquêteur: …………… Dr ENAGUE Annick ……………
2) Nom du contrôleur du questionnaire : ……KALDJOB CHRISTIAN ……………
3) Date de l'entretien: 18../11../2021
4) Nom et N° du Village: N°….. Nom………………………
5) Département: …….Moungo………………
6) Arrondissement : ……………………………Mbanga……….
7) Région :…………………Littoral………
8) Pays :………Cameroun

PARTIE I - CARACTERISATION DU CONSOMMATEUR FINAL

P1.01-Nomet prénom du collecteur
……Serge…NDOUMBE……………………

P1.02 - Sexe 1=……masculin2 = ……féminin

P1.03 - Age☐) ; ☑0 ; 4☐0 ; 5☐) ; ≥☐ (30-40)

P1.04 – **Statut matrimonial** 1 célibataire ; (2) marié(e) ; 3 divorcé ; 4 veuf (ve)

P1.05 Quelle est la taille de votre ménage ? ……4……………………………

P1.06 Quelle est votre religion (1) Chrétiens ; 2 Musulmans ; 3 Autres

Scanné avec CamScanner

P1.07 -**Quel est votre niveau d'instruction?** (1=)....✓.... Cep ; 2= ....Bepc ; 3=...... Bacc ; 4= ....Supérieur.

P1.08-**Quelle est votre activité principale ?** (1=)....✓...... Agriculteur ; 2= .... Éleveur ; 3= ....Commerçant ; 4= ....Fonctionnaire ; 5= .... Artisan ; 6= .... Autres................

P1.09 Quelle est votre activité secondaire ? 1=......Agriculteur ; 2=......Éleveur ; (3=) ..✓..Commerçant ; 4= ......Fonctionnaire ; 5= .... Artisan ; 6= .....Autres.

P1.10- Quelle est votre région d'origine ? ....littoral..............

P1. 11- De quelle ethnie êtes-vous? ....Sawa.........................

P1.12- Depuis combien de temps ramassez-vous des escargots?...........5 ans...................

PARTIE II: COLLECTION D'ESCARGOTS

P2.01- Où ramassez-vous les escargots? (1-) Dans le village✓... ;2- hors du village...

P2.02- Si hors du village, veuillez préciser l'emplacement............✓...................

P2.03- Combien d'escargots collectez-vous en quantités (estimez en terme de seau) ?...01 seau de 15L

P2.04- Au cours de quel mois ramassez-vous le plus d'escargots ?....Août - octobre

P2.05- Veuillez expliquer pourquoi ce mois en particulier ?.....la régularité des pluies en soirée

P2.06- A quelle saison ramassez-vous le plus d'escargots ?...pluvieuse

P2.07- Veuillez expliquerpourquoi cettesaison en particulier?....Espace humide en cette période

P2.08- A quelle heure de la journée ramassez-vous les escargots ?......Nuit

1- Matin....... ; 2- Après-midi......... (3-)La nuit...✓....

P2.09- Veuillez expliquerpourquoi ce temps en particulier ?........c'est la période à laquelle l'escargot sort ....de sa cachette..........pour se nourrir

Scanné avec CamScanner

P2.10- Où collectez-vous le plus ? 1- Autour des maisons………. ; 2- Autour des ordures ……………; 3- Autour des toilettes………… ; 4- Autour des fermes….:……………… ; 5- Dans la forêt……………… ; 6- autres……champs

P2.10- Si dans les petitschamps, quelles cultures les attirent le plus ? 1- Tomate………. ; 2-maïs ………….; 3- Légumes africains…………..; 4-Autres (citez)..banane..

P2.11- Si dans la plantation, quelles cultures les attirent le plus ?

1- Banane…….. ; 2- Cacao…………; 3- Palmier à huile……; 4- Caoutchouc………..; 5- Autres…………………..

P2.12- Pensez-vous que l'utilisation de produits chimiques en agriculture (pesticides, herbicides, engrais) affecte la quantité d'escargots présent dans les champs?

1 - Oui ……2-Non..✓…

P2. 13- -Si oui expliquer ………………/…………………………………………… ..

…………………………………………………………………… ………………………..

P2. 14- Pensez vous qu'il y a autres choses qui pourrait avoir un impact sur la population d'escargot ?

1 - Oui 2-Non

P2. 15- -Si oui lesquelles……les…nématicides…..et…..Molucicide

…………………………………………………………………………………

P2.16- avez-vous pensé élever les escargots ?1 - oui;……2-Non…✓…

P2.17- Si oui, expliquer…………..

P2.18- Si non expliquer…Puisque cela demande un financement et assez de temps

P2.19 avez-vous d'autres commentaire en relation avec le ramassage des escargots……..Non…………

Merci

3
