# Supplementary material for: Harvester perceptions of pesticide impacts on snail collection along Cameroon’s Atlantic coast
Source: PLoS One. 2026 Jun 18;21(6):e0351962. doi: 10.1371/journal.pone.0351962 (PMC13278449; doi:10.1371/journal.pone.0351962)
Supplement: Attachment — Submitted filename: Reviewers Comments-PONE-D-25-23398.docx [file pone.0351962.s004.docx]

**MAJOR CONCERNS**

**1. Clarity of Research Question and Objectives**

The abstract and introduction mention “perceptions of harvesters on pesticide impacts,” but the paper sometimes shifts toward reporting socio-demographic patterns or ecological details without clearly tying them back to pesticides. The objectives need sharper focus. Right now, it feels like two papers mixed: one on socio-economic aspects of snail harvesting and another on pesticide perceptions.

**2. Scientific Rigor and Analysis**

Statistical tests are applied broadly but not always appropriately interpreted. For example:

Reporting a regression model that explains only 6.5% of variance without discussing its weak explanatory power makes the analysis seem overstated.

The Chi-square, ANOVA, and Kruskal-Wallis tests show mostly non-significant results, but the discussion sometimes still draws strong conclusions from them.

The reliance on “perceptions” of pesticide impacts is valuable socially, but the paper sometimes writes as if these perceptions represent ecological reality. The distinction between “belief” and “measured effect” must be consistently clear.

**3. Methodology and Sampling**

The sampling description is confusing. It says “multistage random sampling,” but in practice, it looks more like purposive or stratified site selection. The authors should clarify how participants were recruited and whether the sample is representative.

The description of regression variables (Table 2) is messy, with redundancies and some unclear coding (e.g., “Age in year 1 < 30; 2–30–40; 3–40–50; 3–50–60; 4 ≥ 70” is inconsistent).

**4. Writing Quality and Organization**

The manuscript is verbose, with repetition across sections. For example, the nocturnal activity of snails is mentioned in Methods, Results, and Discussion, each time at length. This could be streamlined.

The figures are referenced but not always well-integrated into the argument. Many simply restate percentages without adding interpretation.

English grammar and phrasing need careful editing. Terms like “snails’ collection” should be revised to “snail collection.”

**5. Novelty and Contribution**

The study is positioned as ecotoxicological but actually relies only on human perceptions. Without direct measurement of pesticide residues or snail health, the claim of “assessing hidden pesticide impacts” feels overstated. The contribution is more socio-cultural than ecological. The authors should reframe the scope honestly.

**MINOR CONCERNS**

Abstract: The phrase “with more involved in nocturnal collection which the most favorable harvesting period” is grammatically incorrect. Needs revision.

Introduction: References to “Achatinadae” should be corrected to Achatinidae throughout.

Methods: Figures and tables should have consistent formatting and professional captions. Right now, some captions read like rough notes.

Results: Percentages sometimes don’t add up or lack denominators (e.g., October recorded 63% of what exactly?). Clarify whether this is proportion of harvesters or harvest volume.

Discussion: Several cultural interpretations (e.g., Toupouri ethnic habits) are interesting but speculative. These need either references or cautious phrasing.

References: Some citations are incomplete (e.g., missing page ranges or inconsistent formatting).


**OVERALL ASSESSMENT**

This manuscript has potential because it looks at an under-studied topic: local knowledge of pesticide impacts on edible snails in Cameroon. The dataset is unique and the issue is relevant for food security and public health.

But right now, the manuscript overreaches in its claims and suffers from methodological and writing issues. To be publishable, it needs:

A tighter framing of objectives.

Clearer and more accurate reporting of statistical results.

A reorientation away from “ecotoxicology” toward “perception and socio-economic study” unless the authors add direct ecological data.

Substantial editing for language, flow, and consistency.
