# Supplementary material for: Harvester perceptions of pesticide impacts on snail collection along Cameroon’s Atlantic coast
Source: PLoS One. 2026 Jun 18;21(6):e0351962. doi: 10.1371/journal.pone.0351962 (PMC13278449; doi:10.1371/journal.pone.0351962)
Supplement: Attachment — Submitted filename: Response to reviewer.docx [file pone.0351962.s005.docx]

**Enangue Njembele Annick Niquaise**
IRAD / Station Specialized in Marine Ecosystems
P.O. Box 219, Kribi, Cameroon
enangueannick@gmail.com

**Kribi, 31 October 2025**

**Subject:** Response Letter - Revised Manuscript Submission

**Dear Editors,**
**PLOS ONE**

We are pleased to resubmit our revised manuscript entitled:
**“Harvester Perceptions of Pesticide Risks in Snail Collection along Cameroon’s Atlantic Coast.”**

We sincerely thank the reviewers and editors for their thorough and constructive feedback, which greatly enhanced the clarity, rigor, and overall presentation of our manuscript. We have carefully revised the manuscript to address all comments point by point.

**Authorship Update**
We kindly request the editor to note the following corrections in authorship order:

- **Ewane** is now listed as the first author, reflecting his primary role in conducting fieldwork and interviews at the main study sites.
- **Enangue Njembele** and **Kaldjob Mbeh** are listed as co-lead authors, having contributed equally to the study’s conceptualization, design, supervision, and manuscript development.
- This change more accurately reflects the intellectual and field contributions of each team member.

**Ethics Statement**
A full Ethics Statement has been added in accordance with PLOS ONE policies:

- Ethical approval details are now explicitly provided.
- Verbal informed consent was obtained from all participants, documented, and witnessed.
- No minors were included in the study, and no personal or identifiable data were collected.
- Additionally, we clarify that the snails studied are common food species in the study area and are not endangered.

**Major Revisions Completed**
In response to reviewer comments, we have made substantial improvements:

1. **Clearer Study Framing**
   - The Introduction and Discussion were revised to emphasize that this is a perception and socio-economic study, rather than an ecotoxicological analysis.
2. **Improved Statistical Interpretation**
   - Non-significant results are reported accurately and interpreted cautiously.
   - Over-interpretation of weak regression results has been removed.
   - The regression model’s low explanatory power (R² = 0.065) is explicitly acknowledged.
3. **Methods and Sampling Clarity**
   - Sampling is now correctly described as purposive recruitment rather than multistage random sampling.
   - Recruitment procedures at markets and within communities are described transparently.
4. **Language, Structure, and Concision**
   - The manuscript, including the title, has been edited for clarity, precision, and reduced redundancy.
   - Repeated ecological descriptions and overly detailed sections were streamlined.
   - Figures and tables were revised to align with PLOS ONE standards.
5. **Integration of Recent Toxicological Evidence**
   - Context on perceptions is strengthened by citing our recent study:
     Enangue Njembele et al. (2025), Journal of Toxicology, on sublethal effects of Cameroon field-used pesticides on Archachatina marginata.
6. **Revised Figure and Table Captions**
   - All captions were rewritten to follow PLOS ONE format, with percentages added where relevant.
   - Figure 18 was removed to avoid unnecessary information.
7. **Updated supporting informations**

Supporting Information files updated and reorganized. An additional supplementary file has been added (S3. Sample of a Completed Questionnaire), and the titles of all supplementary materials (S1, S2, and S3) have been revised to meet PLOS ONE formatting standards. The supplementary files now include: S1. Survey Administered to Snail Harvesters, S2. Minimal Data Set Used for Statistical Analyses, and S3. Sample of a Completed Questionnaire.

8. **Updated Reference List**
   - References were reformatted to comply with ICMJE/Vancouver style, with DOIs added where available.

We believe these revisions have significantly strengthened the manuscript, improving its clarity, methodological transparency, and relevance. We sincerely appreciate the reviewers’ insights and the editor’s guidance and respectfully request the opportunity for reconsideration of our manuscript for publication in **PLOS ONE**.

**Sincerely,**
**Annick N. Enangue Njembele**
Corresponding Author
On behalf of all co-authors
