# Supplementary material for: Harvester perceptions of pesticide impacts on snail collection along Cameroon’s Atlantic coast
Source: PLoS One. 2026 Jun 18;21(6):e0351962. doi: 10.1371/journal.pone.0351962 (PMC13278449; doi:10.1371/journal.pone.0351962)
Supplement: Attachment — Submitted filename: Response to Reviewers.docx [file pone.0351962.s006.docx]

**Enangue Njembele Annick Niquaise**
IRAD / Station Specialized in Marine Ecosystems
P.O. Box 219, Kribi, Cameroon
enangueannick@gmail.com

**Kribi, 19 May 2026**

**Subject:** Response Letter - Revised Manuscript Submission

Dear Editor and Reviewers,

We sincerely thank you for the careful evaluation of our manuscript entitled “Harvester Perceptions of Pesticide Risks in Snail Collection along Cameroon’s Atlantic Coast.” We are grateful for the constructive comments and suggestions, which helped us improve the clarity, consistency, and scientific rigor of the manuscript. Below, we provide a detailed point-by-point response to the comments raised.

# **Responses to Reviewers and Editorial Comments**

## 1. Clarification of the nature of the study (perception-based vs ecological evidence)

### Comment

The manuscript appeared to overinterpret respondents’ perceptions as direct ecological evidence of pesticide impacts.

### Response

We revised the Abstract, Results, Discussion, and Conclusion sections to clearly indicate that the findings are based on harvesters’ perceptions and self-reported experiences rather than direct ecological measurements or pesticide residue analyses.

### Changes made

- Added explicit statements such as:

“These findings reflect local perceptions and self-reported experiences rather than direct ecological measurements.”

- Softened interpretations throughout the Discussion and Conclusion.
- Added recommendations for future ecological monitoring and pesticide residue analyses.

## 2. Clarification of harvesting quantity measurements

### Comment

The harvesting quantity data were unclear, particularly the meaning of values reported for men and women.

### Response

We clarified that the standard field collection unit used by respondents was the 10L bucket and that harvest quantities corresponded to one, two, or three buckets collected per session.

### Changes made

The Results section was revised as follows:

“Most respondents collected one to two 10L buckets of snails per harvesting session, although some harvested up to three buckets. Two-bucket collections were the most common among both men and women.”

We also clarified the gender distribution across harvesting categories.

## 3. Statistical clarification regarding gender differences

### Comment

The manuscript did not clearly indicate whether differences between male and female collectors were statistically significant.

### Response

We performed and reported a chi-square test comparing the proportions of male and female collectors across harvesting categories.

### Changes made

The Results section now states:

“Across all harvesting categories, the proportion of male collectors (70%) was significantly higher than that of female collectors (30%) (χ² test, p < 0.001).”

## 4. Clarification and refinement of figure legends

### Comment

Some figure titles and legends were repetitive or unclear.

### Response

All figure titles and legends were revised for clarity and consistency.

### Changes made

Examples include:

#### Fig 10

“Frequency of snail harvesting according to collection zones.”

#### Fig 11

“Distribution of harvesting quantities across collection zones.”

Legends were simplified to better distinguish:

- harvesting frequency by zone,
- and harvesting quantity according to location.

## 5. Redundancy between figures

### Comment

Certain figures appeared redundant.

### Response

We revised the figure organization and merged complementary figures where appropriate.

### Changes made

Figure 12 now combines two previously separate figures:

- one showing preferred harvesting periods,
- and the second explaining the reasons for night harvesting.

The merged figure was retained because both datasets provide complementary explanations of harvesting behavior.

The revised legend now reads:

“Harvesting occurred predominantly at night (93%), reflecting the nocturnal activity pattern of giant African land snails. Most collectors preferred night harvesting because of increased snail activity and favorable humid conditions.”

## 6. Discussion section interpretation

### Comment

The Discussion required more cautious interpretation of the relationship between pesticide use and snail harvesting outcomes.

### Response

We revised the Discussion to avoid causal interpretations unsupported by the study design.

### Changes made

We clarified that:

- associations observed were weak,
- regression explanatory power was low,
- and other ecological and socio-economic variables may influence harvesting outcomes.

We also distinguished field perceptions from experimentally demonstrated pesticide effects.

## 7. Regression analysis interpretation

### Comment

The manuscript required clearer interpretation of regression outputs.

### Response

The statistical interpretation was revised and simplified.

### Changes made

We now state:

“The low explanatory power of the regression model suggests that additional ecological, environmental, and socio-economic factors not assessed in the present study may influence harvesting outcomes.”

## 8. Ethics statement clarification

### Comment

The Ethics Statement required clarification regarding institutional approval.

### Response

The Ethics Statement was revised to better align with journal expectations for minimal-risk socio-economic surveys.

### Changes made

We clarified that:

- participation was voluntary,
- verbal informed consent was obtained,
- no biological sampling or personal identifiers were collected,
- and formal ethics approval was not required under applicable national regulations.

## 9. Consistency and language corrections

### Comment

Several typographical inconsistencies, formatting problems, and wording issues were present.

### Response

The manuscript was thoroughly revised for language, consistency, and formatting.

### Changes made

Corrections included:

- removal of broken words and hyphenation errors,
- harmonization of terminology,
- correction of figure numbering,
- standardization of seasonal and time-period terminology,
- and refinement of scientific phrasing throughout the manuscript.

## 10. Conclusion section refinement

### Comment

The Conclusion required a more balanced interpretation of the findings.

### Response

The Conclusion was rewritten to better reflect the scope and limitations of the study.

### Changes made

The revised Conclusion now emphasizes:

- the importance of local ecological knowledge,
- the perception-based nature of the findings,
- and the need for future ecological and toxicological studies.

## 11. Abstract revision

### Comment

The Abstract required shortening and improved consistency with the revised manuscript.

### Response

The Abstract was substantially revised.

### Changes made

We:

- reduced redundancy,
- clarified the perception-based nature of the study,
- simplified statistical reporting,
- and aligned the conclusions with the revised Discussion and Results sections.

We sincerely thank the Editor and Reviewers once again for their valuable comments and suggestions, which greatly improved the quality and clarity of the manuscript.

Sincerely,

The Authors
