# Supplementary material for: Harvester perceptions of pesticide impacts on snail collection along Cameroon’s Atlantic coast
Source: PLoS One. 2026 Jun 18;21(6):e0351962. doi: 10.1371/journal.pone.0351962 (PMC13278449; doi:10.1371/journal.pone.0351962)
Supplement: Peer review history

# Peer review: Decision Letter 0

**DOI:** 10.1371/journal.pone.0351962.r001

**Authors:** Shawky M Aboelhadid

18 Sep 2025

PONE-D-25-23398

Assessing Hidden Pesticide Impacts on Achatinadae Snail Harvesting: Harvester Perspectives from Cameroon’s Atlantic agricultural zone

PLOS ONE

Dear Dr. Enangue Njembele,

Thank you for submitting your manuscript to PLOS ONE. After careful consideration, we feel that it has merit but does not fully meet PLOS ONE’s publication criteria as it currently stands. Therefore, we invite you to submit a revised version of the manuscript that addresses the points raised during the review process.

==============================

**ACADEMIC EDITOR:**   The authors should revise the manuscript in accordance to the comments of the reviewers with take care to statistical analysis.

==============================

Please submit your revised manuscript by Nov 01 2025 11:59PM. If you will need more time than this to complete your revisions, please reply to this message or contact the journal office at plosone@plos.org. When you're ready to submit your revision, log on to https://www.editorialmanager.com/pone/ and select the 'Submissions Needing Revision' folder to locate your manuscript file.

Please include the following items when submitting your revised manuscript:

- A rebuttal letter that responds to each point raised by the academic editor and reviewer(s). You should upload this letter as a separate file labeled 'Response to Reviewers'.
- A marked-up copy of your manuscript that highlights changes made to the original version. You should upload this as a separate file labeled 'Revised Manuscript with Track Changes'.
- An unmarked version of your revised paper without tracked changes. You should upload this as a separate file labeled 'Manuscript'.

If you would like to make changes to your financial disclosure, please include your updated statement in your cover letter. Guidelines for resubmitting your figure files are available below the reviewer comments at the end of this letter.

If applicable, we recommend that you deposit your laboratory protocols in protocols.io to enhance the reproducibility of your results. Protocols.io assigns your protocol its own identifier (DOI) so that it can be cited independently in the future. For instructions see: https://journals.plos.org/plosone/s/submission-guidelines#loc-laboratory-protocols. Additionally, PLOS ONE offers an option for publishing peer-reviewed Lab Protocol articles, which describe protocols hosted on protocols.io. Read more information on sharing protocols at https://plos.org/protocols?utm_medium=editorial-email&utm_source=authorletters&utm_campaign=protocols.

We look forward to receiving your revised manuscript.

Kind regards,

Shawky M Aboelhadid, PhD

Academic Editor

PLOS ONE

Journal requirements:

When submitting your revision, we need you to address these additional requirements.

1. Please ensure that your manuscript meets PLOS ONE's style requirements, including those for file naming. The PLOS ONE style templates can be found at

https://journals.plos.org/plosone/s/file?id=wjVg/PLOSOne_formatting_sample_main_body.pdf and

https://journals.plos.org/plosone/s/file?id=ba62/PLOSOne_formatting_sample_title_authors_affiliations.pdf

2. Please provide additional details regarding participant consent. In the ethics statement in the Methods and online submission information, please ensure that you have specified (1) whether consent was informed and (2) what type you obtained (for instance, written or verbal, and if verbal, how it was documented and witnessed). If your study included minors, state whether you obtained consent from parents or guardians. If the need for consent was waived by the ethics committee, please include this information.

If you are reporting a retrospective study of medical records or archived samples, please ensure that you have discussed whether all data were fully anonymized before you accessed them and/or whether the IRB or ethics committee waived the requirement for informed consent. If patients provided informed written consent to have data from their medical records used in research, please include this information.

3. If the reviewer comments include a recommendation to cite specific previously published works, please review and evaluate these publications to determine whether they are relevant and should be cited. There is no requirement to cite these works unless the editor has indicated otherwise.

Additional Editor Comments:

Reviewer #1:

[Note: HTML markup is below. Please do not edit.]

Reviewers' comments:

Reviewer's Responses to Questions

**Comments to the Author**

1. Is the manuscript technically sound, and do the data support the conclusions?

The manuscript must describe a technically sound piece of scientific research with data that supports the conclusions. Experiments must have been conducted rigorously, with appropriate controls, replication, and sample sizes. The conclusions must be drawn appropriately based on the data presented.

Reviewer #1: Partly

**********

2. Has the statistical analysis been performed appropriately and rigorously?

Reviewer #1: No

**********

3. Have the authors made all data underlying the findings in their manuscript fully available?

The PLOS Data policy requires authors to make all data underlying the findings described in their manuscript fully available without restriction, with rare exception (please refer to the Data Availability Statement in the manuscript PDF file). The data should be provided as part of the manuscript or its supporting information, or deposited to a public repository. For example, in addition to summary statistics, the data points behind means, medians and variance measures should be available. If there are restrictions on publicly sharing data—e.g. participant privacy or use of data from a third party—those must be specified.

Reviewer #1: Yes

**********

4. Is the manuscript presented in an intelligible fashion and written in standard English?

PLOS ONE does not copyedit accepted manuscripts, so the language in submitted articles must be clear, correct, and unambiguous. Any typographical or grammatical errors should be corrected at revision, so please note any specific errors here.

Reviewer #1: No

**********

5. Review Comments to the Author

Please use the space provided to explain your answers to the questions above. You may also include additional comments for the author, including concerns about dual publication, research ethics, or publication ethics. (Please upload your review as an attachment if it exceeds 20,000 characters)

Reviewer #1: MAJOR CONCERNS

1. Clarity of Research Question and Objectives

The abstract and introduction mention “perceptions of harvesters on pesticide impacts,” but the paper sometimes shifts toward reporting socio-demographic patterns or ecological details without clearly tying them back to pesticides. The objectives need sharper focus. Right now, it feels like two papers mixed: one on socio-economic aspects of snail harvesting and another on pesticide perceptions.

2. Scientific Rigor and Analysis

Statistical tests are applied broadly but not always appropriately interpreted. For example:

Reporting a regression model that explains only 6.5% of variance without discussing its weak explanatory power makes the analysis seem overstated.

The Chi-square, ANOVA, and Kruskal-Wallis tests show mostly non-significant results, but the discussion sometimes still draws strong conclusions from them.

The reliance on “perceptions” of pesticide impacts is valuable socially, but the paper sometimes writes as if these perceptions represent ecological reality. The distinction between “belief” and “measured effect” must be consistently clear.

3. Methodology and Sampling

The sampling description is confusing. It says “multistage random sampling,” but in practice, it looks more like purposive or stratified site selection. The authors should clarify how participants were recruited and whether the sample is representative.

The description of regression variables (Table 2) is messy, with redundancies and some unclear coding (e.g., “Age in year 1 < 30; 2–30–40; 3–40–50; 3–50–60; 4 ≥ 70” is inconsistent).

4. Writing Quality and Organization

The manuscript is verbose, with repetition across sections. For example, the nocturnal activity of snails is mentioned in Methods, Results, and Discussion, each time at length. This could be streamlined.

The figures are referenced but not always well-integrated into the argument. Many simply restate percentages without adding interpretation.

English grammar and phrasing need careful editing. Terms like “snails’ collection” should be revised to “snail collection.”

5. Novelty and Contribution

The study is positioned as ecotoxicological but actually relies only on human perceptions. Without direct measurement of pesticide residues or snail health, the claim of “assessing hidden pesticide impacts” feels overstated. The contribution is more socio-cultural than ecological. The authors should reframe the scope honestly.

MINOR CONCERNS

Abstract: The phrase “with more involved in nocturnal collection which the most favorable harvesting period” is grammatically incorrect. Needs revision.

Introduction: References to “Achatinadae” should be corrected to Achatinidae throughout.

Methods: Figures and tables should have consistent formatting and professional captions. Right now, some captions read like rough notes.

Results: Percentages sometimes don’t add up or lack denominators (e.g., October recorded 63% of what exactly?). Clarify whether this is proportion of harvesters or harvest volume.

Discussion: Several cultural interpretations (e.g., Toupouri ethnic habits) are interesting but speculative. These need either references or cautious phrasing.

References: Some citations are incomplete (e.g., missing page ranges or inconsistent formatting).

OVERALL ASSESSMENT

This manuscript has potential because it looks at an under-studied topic: local knowledge of pesticide impacts on edible snails in Cameroon. The dataset is unique and the issue is relevant for food security and public health.

But right now, the manuscript overreaches in its claims and suffers from methodological and writing issues. To be publishable, it needs:

A tighter framing of objectives.

Clearer and more accurate reporting of statistical results.

A reorientation away from “ecotoxicology” toward “perception and socio-economic study” unless the authors add direct ecological data.

Substantial editing for language, flow, and consistency.

**********

6. PLOS authors have the option to publish the peer review history of their article (what does this mean?). If published, this will include your full peer review and any attached files.

If you choose “no”, your identity will remain anonymous but your review may still be made public.

**Do you want your identity to be public for this peer review?**   For information about this choice, including consent withdrawal, please see our Privacy Policy.

Reviewer #1: **Yes:**   Dr. Mohammad Wasim Aktar

**********

[NOTE: If reviewer comments were submitted as an attachment file, they will be attached to this email and accessible via the submission site. Please log into your account, locate the manuscript record, and check for the action link "View Attachments". If this link does not appear, there are no attachment files.]

While revising your submission, please upload your figure files to the Preflight Analysis and Conversion Engine (PACE) digital diagnostic tool, https://pacev2.apexcovantage.com/. PACE helps ensure that figures meet PLOS requirements. To use PACE, you must first register as a user. Registration is free. Then, login and navigate to the UPLOAD tab, where you will find detailed instructions on how to use the tool. If you encounter any issues or have any questions when using PACE, please email PLOS at figures@plos.org. Please note that Supporting Information files do not need this step.

Attachment Submitted filename: Reviewers Comments-PONE-D-25-23398.docx

# Author comment: Author response to Decision Letter 1

**DOI:** 10.1371/journal.pone.0351962.r002

20 Nov 2025

Enangue Njembele Annick Niquaise

IRAD / Station Specialized in Marine Ecosystems

P.O. Box 219, Kribi, Cameroon

enangueannick@gmail.com

Kribi, 31 October 2025

Subject: Response Letter - Revised Manuscript Submission

Dear Editors,

PLOS ONE

We are pleased to resubmit our revised manuscript entitled:

“Harvester Perceptions of Pesticide Risks in Snail Collection along Cameroon’s Atlantic Coast.”

We sincerely thank the reviewers and editors for their thorough and constructive feedback, which greatly enhanced the clarity, rigor, and overall presentation of our manuscript. We have carefully revised the manuscript to address all comments point by point.

Authorship Update

We kindly request the editor to note the following corrections in authorship order:

• Ewane is now listed as the first author, reflecting his primary role in conducting fieldwork and interviews at the main study sites.

• Enangue Njembele and Kaldjob Mbeh are listed as co-lead authors, having contributed equally to the study’s conceptualization, design, supervision, and manuscript development.

• This change more accurately reflects the intellectual and field contributions of each team member.

Ethics Statement

A full Ethics Statement has been added in accordance with PLOS ONE policies:

• Ethical approval details are now explicitly provided.

• Verbal informed consent was obtained from all participants, documented, and witnessed.

• No minors were included in the study, and no personal or identifiable data were collected.

• Additionally, we clarify that the snails studied are common food species in the study area and are not endangered.

Major Revisions Completed

In response to reviewer comments, we have made substantial improvements:

1. Clearer Study Framing

o The Introduction and Discussion were revised to emphasize that this is a perception and socio-economic study, rather than an ecotoxicological analysis.

2. Improved Statistical Interpretation

o Non-significant results are reported accurately and interpreted cautiously.

o Over-interpretation of weak regression results has been removed.

o The regression model’s low explanatory power (R² = 0.065) is explicitly acknowledged.

3. Methods and Sampling Clarity

o Sampling is now correctly described as purposive recruitment rather than multistage random sampling.

o Recruitment procedures at markets and within communities are described transparently.

4. Language, Structure, and Concision

o The manuscript, including the title, has been edited for clarity, precision, and reduced redundancy.

o Repeated ecological descriptions and overly detailed sections were streamlined.

o Figures and tables were revised to align with PLOS ONE standards.

5. Integration of Recent Toxicological Evidence

o Context on perceptions is strengthened by citing our recent study:

Enangue Njembele et al. (2025), Journal of Toxicology, on sublethal effects of Cameroon field-used pesticides on Archachatina marginata.

6. Revised Figure and Table Captions

o All captions were rewritten to follow PLOS ONE format, with percentages added where relevant.

o Figure 18 was removed to avoid unnecessary information.

7. Updated Reference List

o References were reformatted to comply with ICMJE/Vancouver style, with DOIs added where available.

We believe these revisions have significantly strengthened the manuscript, improving its clarity, methodological transparency, and relevance. We sincerely appreciate the reviewers’ insights and the editor’s guidance and respectfully request the opportunity for reconsideration of our manuscript for publication in PLOS ONE.

Sincerely,

Annick N. Enangue Njembele

Corresponding Author

On behalf of all co-authors

Attachment Submitted filename: Response to reviewer.docx

# Peer review: Decision Letter 1

**DOI:** 10.1371/journal.pone.0351962.r003

**Authors:** Xiaoshan M Zhu

1 May 2026

PONE-D-25-23398R1

Harvester Perceptions of Pesticide Risks in Snail Collection along Cameroon’s Atlantic Coast

PLOS One

Dear Dr. Enangue Njembele,

Thank you for submitting your manuscript to PLOS ONE. After careful consideration, we feel that it has merit but does not fully meet PLOS ONE’s publication criteria as it currently stands. Therefore, we invite you to submit a revised version of the manuscript that addresses the points raised during the review process.

Please submit your revised manuscript by Jun 15 2026 11:59PM. If you will need more time than this to complete your revisions, please reply to this message or contact the journal office at plosone@plos.org. When you're ready to submit your revision, log on to https://www.editorialmanager.com/pone/ and select the 'Submissions Needing Revision' folder to locate your manuscript file.

Please include the following items when submitting your revised manuscript:

- A letter that responds to each point raised by the academic editor and reviewer(s). You should upload this letter as a separate file labeled 'Response to Reviewers'.
- A marked-up copy of your manuscript that highlights changes made to the original version. You should upload this as a separate file labeled 'Revised Manuscript with Track Changes'.
- An unmarked version of your revised paper without tracked changes. You should upload this as a separate file labeled 'Manuscript'.

If you would like to make changes to your financial disclosure, please include your updated statement in your cover letter. Guidelines for resubmitting your figure files are available below the reviewer comments at the end of this letter.

If applicable, we recommend that you deposit your laboratory protocols in protocols.io to enhance the reproducibility of your results. Protocols.io assigns your protocol its own identifier (DOI) so that it can be cited independently in the future. For instructions see: https://journals.plos.org/plosone/s/submission-guidelines#loc-laboratory-protocols. Additionally, PLOS ONE offers an option for publishing peer-reviewed Lab Protocol articles, which describe protocols hosted on protocols.io. Read more information on sharing protocols at https://plos.org/protocols?utm_medium=editorial-email&utm_source=authorletters&utm_campaign=protocols.

As the corresponding author, your ORCID iD is verified in the submission system and will appear in the published article. PLOS supports the use of ORCID, and we encourage all coauthors to register for an ORCID iD and use it as well. Please encourage your coauthors to verify their ORCID iD within the submission system before final acceptance, as unverified ORCID iDs will not appear in the published article. *Only*   the individual author can complete the verification step; PLOS staff *cannot*   verify ORCID iDs on behalf of authors.

We look forward to receiving your revised manuscript.

Kind regards,

Xiaoshan Zhu, Ph.D.

Academic Editor

PLOS One

Journal Requirements:

1. If the reviewer comments include a recommendation to cite specific previously published works, please review and evaluate these publications to determine whether they are relevant and should be cited. There is no requirement to cite these works unless the editor has indicated otherwise.

2. Please review your reference list to ensure that it is complete and correct. If you have cited papers that have been retracted, please include the rationale for doing so in the manuscript text, or remove these references and replace them with relevant current references. Any changes to the reference list should be mentioned in the rebuttal letter that accompanies your revised manuscript. If you need to cite a retracted article, indicate the article’s retracted status in the References list and also include a citation and full reference for the retraction notice.

[Note: HTML markup is below. Please do not edit.]

Reviewers' comments:

Reviewer's Responses to Questions

**Comments to the Author**

1. If the authors have adequately addressed your comments raised in a previous round of review and you feel that this manuscript is now acceptable for publication, you may indicate that here to bypass the “Comments to the Author” section, enter your conflict of interest statement in the “Confidential to Editor” section, and submit your "Accept" recommendation.

Reviewer #2: All comments have been addressed

Reviewer #3: (No Response)

**********

2. Is the manuscript technically sound, and do the data support the conclusions?

The manuscript must describe a technically sound piece of scientific research with data that supports the conclusions. Experiments must have been conducted rigorously, with appropriate controls, replication, and sample sizes. The conclusions must be drawn appropriately based on the data presented.

Reviewer #2: Yes

Reviewer #3: Yes

**********

3. Has the statistical analysis been performed appropriately and rigorously?

Reviewer #2: Yes

Reviewer #3: Yes

**********

4. Have the authors made all data underlying the findings in their manuscript fully available?

The PLOS Data policy requires authors to make all data underlying the findings described in their manuscript fully available without restriction, with rare exception (please refer to the Data Availability Statement in the manuscript PDF file). The data should be provided as part of the manuscript or its supporting information, or deposited to a public repository. For example, in addition to summary statistics, the data points behind means, medians and variance measures should be available. If there are restrictions on publicly sharing data—e.g. participant privacy or use of data from a third party—those must be specified.

Reviewer #2: Yes

Reviewer #3: Yes

**********

5. Is the manuscript presented in an intelligible fashion and written in standard English?

PLOS ONE does not copyedit accepted manuscripts, so the language in submitted articles must be clear, correct, and unambiguous. Any typographical or grammatical errors should be corrected at revision, so please note any specific errors here.

Reviewer #2: Yes

Reviewer #3: Yes

**********

6. Review Comments to the Author

Please use the space provided to explain your answers to the questions above. You may also include additional comments for the author, including concerns about dual publication, research ethics, or publication ethics. (Please upload your review as an attachment if it exceeds 20,000 characters)

Reviewer #2: This introduction effectively situates the study within a relevant and interdisciplinary context, linking pesticide use along Cameroon’s Atlantic coast to potential ecological and public health risks for snail harvesters. The authors make a strong case for examining harvesters’ perceptions as an early warning tool where formal monitoring is absent. However, the introduction suffers from several conceptual and logical weaknesses that require minor revision. First, “perception” is not defined or operationalised, leaving it unclear whether the study refers to awareness of pesticide presence, observed snail declines, or health concerns. Second, the argument moves too directly from “pesticides harm snails” to “harvesters should perceive risks” without considering mediating factors such as visibility of contamination or local knowledge. Third, the research gap is stated vaguely (“limited monitoring”) and could be sharpened by clarifying that no previous perception-based study exists for snails in this region. Finally, key terms such as “harvesters” (e.g., subsistence vs. commercial collectors) and the sociopolitical unrest driving migration deserve brief specification.

Reviewer #3: General comments

The revised manuscript has been improved and is now more clearly framed as a perception-based socio-economic study rather than a direct ecotoxicological assessment. The topic is relevant and provides useful local information on pesticide risk perception, snail harvesting practices, and sustainable resource use along Cameroon’s Atlantic coast. Overall, the manuscript may be considered for publication after minor revision.

Specific comments

1. Please ensure consistency between the ethics statement in the submission form and that in the manuscript, especially since the study involved interviews with adult participants and verbal informed consent.

2. The sampling and recruitment procedure should be described a little more clearly, including the distinction between market-based recruitment and community nomination, as well as the potential limitation of purposive sampling.

3. The statistical analysis section could be simplified and clarified. Please specify which statistical tests correspond to which research questions and avoid overemphasizing weak associations, particularly given the low explanatory power of the regression model.

4. Please carefully check all figure numbers, captions, and in-text references to ensure consistency and avoid mismatches.

5. The Discussion and Conclusion should continue to clearly state that the findings are based on harvesters’ perceptions and self-reported experiences, not on direct measurements of pesticide residues, snail abundance, or ecological toxicity.

**********

7. PLOS authors have the option to publish the peer review history of their article (what does this mean?). If published, this will include your full peer review and any attached files.

If you choose “no”, your identity will remain anonymous but your review may still be made public.

**Do you want your identity to be public for this peer review?**   For information about this choice, including consent withdrawal, please see our Privacy Policy.

Reviewer #2: No

Reviewer #3: No

**********

[NOTE: If reviewer comments were submitted as an attachment file, they will be attached to this email and accessible via the submission site. Please log into your account, locate the manuscript record, and check for the action link "View Attachments". If this link does not appear, there are no attachment files.]

To ensure your figures meet our technical requirements, please review our figure guidelines: https://journals.plos.org/plosone/s/figures

You may also use PLOS’s free figure tool, NAAS, to help you prepare publication quality figures: https://journals.plos.org/plosone/s/figures#loc-tools-for-figure-preparation.

NAAS will assess whether your figures meet our technical requirements by comparing each figure against our figure specifications.

# Author comment: Author response to Decision Letter 2

**DOI:** 10.1371/journal.pone.0351962.r004

19 May 2026

Enangue Njembele Annick Niquaise

IRAD / Station Specialized in Marine Ecosystems

P.O. Box 219, Kribi, Cameroon

enangueannick@gmail.com

Kribi, 19 May 2026

Subject: Response Letter - Revised Manuscript Submission

Dear Editor and Reviewers,

We sincerely thank you for the careful evaluation of our manuscript entitled “Harvester Perceptions of Pesticide Risks in Snail Collection along Cameroon’s Atlantic Coast.” We are grateful for the constructive comments and suggestions, which helped us improve the clarity, consistency, and scientific rigor of the manuscript. Below, we provide a detailed point-by-point response to the comments raised.

Responses to Reviewers and Editorial Comments

1. Clarification of the nature of the study (perception-based vs ecological evidence)

Comment

The manuscript appeared to overinterpret respondents’ perceptions as direct ecological evidence of pesticide impacts.

Response

We revised the Abstract, Results, Discussion, and Conclusion sections to clearly indicate that the findings are based on harvesters’ perceptions and self-reported experiences rather than direct ecological measurements or pesticide residue analyses.

Changes made

• Added explicit statements such as:

“These findings reflect local perceptions and self-reported experiences rather than direct ecological measurements.”

• Softened interpretations throughout the Discussion and Conclusion.

• Added recommendations for future ecological monitoring and pesticide residue analyses.

2. Clarification of harvesting quantity measurements

Comment

The harvesting quantity data were unclear, particularly the meaning of values reported for men and women.

Response

We clarified that the standard field collection unit used by respondents was the 10L bucket and that harvest quantities corresponded to one, two, or three buckets collected per session.

Changes made

The Results section was revised as follows:

“Most respondents collected one to two 10L buckets of snails per harvesting session, although some harvested up to three buckets. Two-bucket collections were the most common among both men and women.”

We also clarified the gender distribution across harvesting categories.

3. Statistical clarification regarding gender differences

Comment

The manuscript did not clearly indicate whether differences between male and female collectors were statistically significant.

Response

We performed and reported a chi-square test comparing the proportions of male and female collectors across harvesting categories.

Changes made

The Results section now states:

“Across all harvesting categories, the proportion of male collectors (70%) was significantly higher than that of female collectors (30%) (χ² test, p < 0.001).”

4. Clarification and refinement of figure legends

Comment

Some figure titles and legends were repetitive or unclear.

Response

All figure titles and legends were revised for clarity and consistency.

Changes made

Examples include:

Fig 10

“Frequency of snail harvesting according to collection zones.”

Fig 11

“Distribution of harvesting quantities across collection zones.”

Legends were simplified to better distinguish:

• harvesting frequency by zone,

• and harvesting quantity according to location.

5. Redundancy between figures

Comment

Certain figures appeared redundant.

Response

We revised the figure organization and merged complementary figures where appropriate.

Changes made

Figure 12 now combines two previously separate figures:

• one showing preferred harvesting periods,

• and the second explaining the reasons for night harvesting.

The merged figure was retained because both datasets provide complementary explanations of harvesting behavior.

The revised legend now reads:

“Harvesting occurred predominantly at night (93%), reflecting the nocturnal activity pattern of giant African land snails. Most collectors preferred night harvesting because of increased snail activity and favorable humid conditions.”

6. Discussion section interpretation

Comment

The Discussion required more cautious interpretation of the relationship between pesticide use and snail harvesting outcomes.

Response

We revised the Discussion to avoid causal interpretations unsupported by the study design.

Changes made

We clarified that:

• associations observed were weak,

• regression explanatory power was low,

• and other ecological and socio-economic variables may influence harvesting outcomes.

We also distinguished field perceptions from experimentally demonstrated pesticide effects.

7. Regression analysis interpretation

Comment

The manuscript required clearer interpretation of regression outputs.

Response

The statistical interpretation was revised and simplified.

Changes made

We now state:

“The low explanatory power of the regression model suggests that additional ecological, environmental, and socio-economic factors not assessed in the present study may influence harvesting outcomes.”

8. Ethics statement clarification

Comment

The Ethics Statement required clarification regarding institutional approval.

Response

The Ethics Statement was revised to better align with journal expectations for minimal-risk socio-economic surveys.

Changes made

We clarified that:

• participation was voluntary,

• verbal informed consent was obtained,

• no biological sampling or personal identifiers were collected,

• and formal ethics approval was not required under applicable national regulations.

9. Consistency and language corrections

Comment

Several typographical inconsistencies, formatting problems, and wording issues were present.

Response

The manuscript was thoroughly revised for language, consistency, and formatting.

Changes made

Corrections included:

• removal of broken words and hyphenation errors,

• harmonization of terminology,

• correction of figure numbering,

• standardization of seasonal and time-period terminology,

• and refinement of scientific phrasing throughout the manuscript.

10. Conclusion section refinement

Comment

The Conclusion required a more balanced interpretation of the findings.

Response

The Conclusion was rewritten to better reflect the scope and limitations of the study.

Changes made

The revised Conclusion now emphasizes:

• the importance of local ecological knowledge,

• the perception-based nature of the findings,

• and the need for future ecological and toxicological studies.

11. Abstract revision

Comment

The Abstract required shortening and improved consistency with the revised manuscript.

Response

The Abstract was substantially revised.

Changes made

We:

• reduced redundancy,

• clarified the perception-based nature of the study,

• simplified statistical reporting,

• and aligned the conclusions with the revised Discussion and Results sections.

We sincerely thank the Editor and Reviewers once again for their valuable comments and suggestions, which greatly improved the quality and clarity of the manuscript.

Sincerely,

The Authors

Attachment Submitted filename: Response to Reviewers.docx

# Peer review: Decision Letter 2

**DOI:** 10.1371/journal.pone.0351962.r005

**Authors:** Shawky M Aboelhadid

3 Jun 2026

<p>Harvester Perceptions of Pesticide Risks in Snail Collection along Cameroon’s Atlantic Coast

PONE-D-25-23398R2

Dear Dr. Enangue Njembele,

We’re pleased to inform you that your manuscript has been judged scientifically suitable for publication and will be formally accepted for publication once it meets all outstanding technical requirements.

Within one week, you’ll receive an e-mail detailing the required amendments. When these have been addressed, you’ll receive a formal acceptance letter and your manuscript will be scheduled for publication.

An invoice will be generated when your article is formally accepted. Please note, if your institution has a publishing partnership with PLOS and your article meets the relevant criteria, all or part of your publication costs will be covered. Please make sure your user information is up-to-date by logging into Editorial Manager at Editorial Manager® and clicking the ‘Update My Information' link at the top of the page. For questions related to billing, please contact billing support.

If your institution or institutions have a press office, please notify them about your upcoming paper to help maximize its impact. If they’ll be preparing press materials, please inform our press team as soon as possible -- no later than 48 hours after receiving the formal acceptance. Your manuscript will remain under strict press embargo until 2 pm Eastern Time on the date of publication. For more information, please contact onepress@plos.org.

Kind regards,

Xiaoshan Zhu, Ph.D.

Academic Editor

PLOS One

Additional Editor Comments (optional):

Reviewers' comments:

Reviewer's Responses to Questions

**Comments to the Author**

1. If the authors have adequately addressed your comments raised in a previous round of review and you feel that this manuscript is now acceptable for publication, you may indicate that here to bypass the “Comments to the Author” section, enter your conflict of interest statement in the “Confidential to Editor” section, and submit your "Accept" recommendation.

Reviewer #2: All comments have been addressed

Reviewer #3: (No Response)

**********

2. Is the manuscript technically sound, and do the data support the conclusions?

The manuscript must describe a technically sound piece of scientific research with data that supports the conclusions. Experiments must have been conducted rigorously, with appropriate controls, replication, and sample sizes. The conclusions must be drawn appropriately based on the data presented.

Reviewer #2: Yes

Reviewer #3: (No Response)

**********

3. Has the statistical analysis been performed appropriately and rigorously?

Reviewer #2: Yes

Reviewer #3: (No Response)

**********

4. Have the authors made all data underlying the findings in their manuscript fully available?

The PLOS Data policy requires authors to make all data underlying the findings described in their manuscript fully available without restriction, with rare exception (please refer to the Data Availability Statement in the manuscript PDF file). The data should be provided as part of the manuscript or its supporting information, or deposited to a public repository. For example, in addition to summary statistics, the data points behind means, medians and variance measures should be available. If there are restrictions on publicly sharing data—e.g. participant privacy or use of data from a third party—those must be specified.

Reviewer #2: Yes

Reviewer #3: (No Response)

**********

5. Is the manuscript presented in an intelligible fashion and written in standard English?

PLOS ONE does not copyedit accepted manuscripts, so the language in submitted articles must be clear, correct, and unambiguous. Any typographical or grammatical errors should be corrected at revision, so please note any specific errors here.

Reviewer #2: Yes

Reviewer #3: (No Response)

**********

6. Review Comments to the Author

Please use the space provided to explain your answers to the questions above. You may also include additional comments for the author, including concerns about dual publication, research ethics, or publication ethics. (Please upload your review as an attachment if it exceeds 20,000 characters)

Reviewer #2: The authors have addressed all my previous concerns thoroughly in the revised manuscript. I have no further comments or suggestions.

Reviewer #3: (No Response)

**********

7. PLOS authors have the option to publish the peer review history of their article (what does this mean?). If published, this will include your full peer review and any attached files.

If you choose “no”, your identity will remain anonymous but your review may still be made public.

**Do you want your identity to be public for this peer review?**   For information about this choice, including consent withdrawal, please see our Privacy Policy.

Reviewer #2: No

Reviewer #3: No

**********

# Editor report: Acceptance letter

**DOI:** 10.1371/journal.pone.0351962.r006

**Authors:** Shawky M Aboelhadid

PONE-D-25-23398R2

PLOS One

Dear Dr. Enangue Njembele,

I'm pleased to inform you that your manuscript has been deemed suitable for publication in PLOS One. Congratulations! Your manuscript is now being handed over to our production team.

At this stage, our production department will prepare your paper for publication. This includes ensuring the following:

* All references, tables, and figures are properly cited

* All relevant supporting information is included in the manuscript submission,

* There are no issues that prevent the paper from being properly typeset

You will receive further instructions from the production team, including instructions on how to review your proof when it is ready. Please keep in mind that we are working through a large volume of accepted articles, so please give us a few days to review your paper and let you know the next and final steps.

Lastly, if your institution or institutions have a press office, please let them know about your upcoming paper now to help maximize its impact. If they'll be preparing press materials, please inform our press team within the next 48 hours. Your manuscript will remain under strict press embargo until 2 pm Eastern Time on the date of publication. For more information, please contact onepress@plos.org.

You will receive an invoice from PLOS for your publication fee after your manuscript has reached the completed accept phase. If you receive an email requesting payment before acceptance or for any other service, this may be a phishing scheme. Learn how to identify phishing emails and protect your accounts at https://explore.plos.org/phishing.

If we can help with anything else, please email us at customercare@plos.org.

Thank you for submitting your work to PLOS ONE and supporting open access.

Kind regards,

PLOS ONE Editorial Office Staff

on behalf of

Dr. Xiaoshan Zhu

Academic Editor

PLOS One
